# Interferon-inducible ribonuclease ISG20 inhibits hepatitis B virus replication through directly binding to the epsilon stem-loop structure of viral RNA

**DOI:** 10.1371/journal.ppat.1006296

**Published:** 2017-04-11

**Authors:** Yuanjie Liu, Hui Nie, Richeng Mao, Bidisha Mitra, Dawei Cai, Ran Yan, Ju-Tao Guo, Timothy M. Block, Nadir Mechti, Haitao Guo

**Affiliations:** 1 Department of Microbiology and Immunology, Indiana University School of Medicine, Indianapolis, Indiana, United States of America; 2 Department of Microbiology and Immunology, Drexel University College of Medicine, Philadelphia, Pennsylvania, United States of America; 3 Baruch S. Blumberg Institute, Doylestown, Pennsylvania, United States of America; 4 CNRS, UMR5235, DIMNP, University of Montpellier 2, Montpellier, France; University of California, San Diego, UNITED STATES

## Abstract

Hepatitis B virus (HBV) replicates its DNA genome through reverse transcription of a viral RNA pregenome. We report herein that the interferon (IFN) stimulated exoribonuclease gene of 20 KD (ISG20) inhibits HBV replication through degradation of HBV RNA. ISG20 expression was observed at basal level and was highly upregulated upon IFN treatment in hepatocytes, and knock down of ISG20 resulted in elevation of HBV replication and attenuation of IFN-mediated antiviral effect. The sequence element conferring the susceptibility of HBV RNA to ISG20-mediated RNA degradation was mapped at the HBV RNA terminal redundant region containing epsilon (ε) stem-loop. Furthermore, ISG20-induced HBV RNA degradation relies on its ribonuclease activity, as the enzymatic inactive form ISG20^D94G^ was unable to promote HBV RNA decay. Interestingly, ISG20^D94G^ retained antiviral activity against HBV DNA replication by preventing pgRNA encapsidation, resulting from a consequence of ISG20-ε interaction. This interaction was further characterized by *in vitro* electrophoretic mobility shift assay (EMSA) and ISG20 was able to bind HBV ε directly in absence of any other cellular proteins, indicating a direct ε RNA binding capability of ISG20; however, cofactor(s) may be required for ISG20 to efficiently degrade ε. In addition, the lower stem portion of ε is the major ISG20 binding site, and the removal of 4 base pairs from the bottom portion of ε abrogated the sensitivity of HBV RNA to ISG20, suggesting that the specificity of ISG20-ε interaction relies on both RNA structure and sequence. Furthermore, the C-terminal Exonuclease III (ExoIII) domain of ISG20 was determined to be responsible for interacting with ε, as the deletion of ExoIII abolished *in vitro* ISG20-ε binding and intracellular HBV RNA degradation. Taken together, our study sheds light on the underlying mechanisms of IFN-mediated HBV inhibition and the antiviral mechanism of ISG20 in general.

## Author summary

HBV is a DNA virus but replicates its DNA *via* retrotranscription of a viral RNA pregenome. ISG20, an antiviral RNase induced by interferons, inhibits the replication of many RNA viruses but the underlying molecular antiviral mechanism remains elusive. Since all the known viruses, except for prions, have RNA products in their life cycles, ISG20 can be a broad spectrum antiviral protein; but in order to distinguish viral RNA from host RNA, ISG20 may have evolved to recognize virus-specific signals as its antiviral target. We demonstrated herein that ISG20 selectively binds to a unique stem-loop structure called epsilon (ε) in all HBV RNA species and degrades viral RNA to inhibit HBV replication. Because ε is the HBV pregenomic RNA packaging signal and reverse transcription priming site, the binding of ISG20 to ε, even in the absence of ribonuclease activity, results in antiviral effect to prevent DNA replication due to preventing viral polymerase binding to pgRNA. We also determined the structure and sequence requirements of ε RNA and ISG20 protein for ISG20-ε binding and antiviral activity. Such information will aid the function study of ISG20 against viral pathogens in host innate defense, and ISG20 has potentials to be developed into a therapeutic agent for viral diseases including hepatitis B.

## Introduction

Hepatitis B virus (HBV) infection remains a significant health threat to humans, leading to the elevated rate of severe liver diseases, such as fulminant hepatitis, fibrosis, cirrhosis, primary hepatocellular carcinoma, and other clinical complications [1].

HBV is the prototype member of *hepadnaviridae* family which contains a number of DNA viruses replicating their genome through reverse transcription of a viral RNA intermediate in hepatocytes. After cell infection mediated by the viral receptor, called sodium taurocholate cotransporting polypeptide (NTCP) [2], the 3.2kb relaxed circular (rc) viral DNA genome in the virion enters the nucleus and transforms into a nucleosome-decorated covalently closed circular (ccc) DNA minichromosome. Using cccDNA as transcription template, five viral mRNAs are transcribed from different transcription initiation sites but all terminated at a same polyadenylation site, which are, 3.5~3.6 kb precore mRNA, 3.5 kb pregenomic (pg) RNA, 2.4 and 2.1 kb surface (envelope) mRNA, and 0.7kb X mRNA. Besides serving as the template for translation of viral core protein and polymerase (pol), the pgRNA is also the template for reverse transcription. The pol recognizes a stem loop structure (epsilon, ε) at the 5’ terminus of pgRNA to recruit core proteins to encapsidate pol/pgRNA complex into nucleocapsid, where the reverse transcription takes place to yield progeny viral rcDNA [3]. Considering the importance of viral RNA in HBV life cycle, it is conceivable that HBV RNA reduction will result in a suppression of both viral DNA replication and antigen production. Therefore, hijacking HBV RNA is believed to be an important antiviral strategy for host defense against HBV. In line with this, interferons are able to directly reduce HBV RNA through both transcriptional and posttranscriptional mechanisms [4–7]. In terms of interferon-stimulated genes (ISG), the tripartite motif-containing protein 22 (TRIM22) and DEAD-box RNA helicase DDX3 have been shown to inhibit HBV RNA transcription [8, 9], and the zinc finger antiviral protein (ZAP) and myeloid differentiation primary response gene 88 (MyD88) have been identified as host intrinsic antiviral factors against HBV through promoting the decay of HBV RNA [10, 11].

In search of ISGs that inhibit HBV replication, we have previously found that the 20 KDa interferon-stimulated gene product, referred to as ISG20, inhibited HBV replication primarily through reducing HBV RNA transcript levels in cell cultures [4]. This protein, which has been shown to be induced by both type I and type II IFNs, belongs to DEDDh subgroup of the DEDD exonuclease superfamily. Sequence homologies are distributed at three distinct motifs termed as ExoI (a.a7-16), ExoII (a.a 86–101) and ExoIII (a.a 147–157) [12]. ISG20 is a 3’ to 5’ exonuclease with the substrate preference of single-stranded RNA over double-stranded RNA or single-stranded DNA. Its enzymatic activity is mediated by the ExoII motif, as substitution of the Aspartic acid 94 (D94) in the ExoII motif to Glycine (D94G) completely abolishes its exonuclease activity [13]. Cellular proteins may be involved in regulation of ISG20 in substrate recognition and/or binding in order to avoid any non-specific cell toxicity, however which remain to be identified.

ISG20 has been shown playing an important role in host antiviral innate immune defense [14]. It is a key ISG against alphavirus replication in host antiviral defense mediated via IFN-α/β signaling [15]. Cells with overexpressed ISG20 are resistant to the infections of RNA viruses including vesicular stomatitis virus (VSV), influenza virus, encephalomyocarditis virus (EMCV), hepatitis A and C virus (HAV and HCV), yellow fever virus, and HIV, but not to a DNA virus, adenovirus [16–18].

In the context of HBV infection, it has been reported that ISG20 was highly induced during IFN-mediated suppression of viral replication in HBV transgenic mouse hepatocyte cell lines [19], and in the liver of acutely HBV infected Chimpanzee during viral clearance phase [20]. Interestingly, chronic hepatitis B patients who had good responses to IFN treatment showed significantly higher levels of ISG20 in their livers compared to either the patients who were non-responders to IFN treatment or the healthy controls [21]. All the above findings suggest that ISG20 may play a role in suppressing HBV in response to host immune activation or IFN-based immunotherapy.

Therefore, we set out to further characterize the antiviral function and mechanism of ISG20 in innate control of HBV. We report herein that: 1) the endogenous ISG20 is a restriction factor for HBV replication and it can be upregulated by all three types of IFN to further inhibit HBV; 2) ISG20 inhibits HBV replication primarily by promoting viral RNA degradation; 3) ISG20-mediated HBV RNA degradation depends on its ribonuclease activity, but the enzymatically inactivated form retains antiviral activity against pgRNA encapsidation by competing with HBV pol for pgRNA binding; 4) ISG20 recognizes HBV RNA through directly binding to the lower stem region of epsilon and the binding domain on ISG20 is located in Exo III motif; 5) ISG20-epsilon binding is essential to ISG20-mediated HBV RNA degradation in cell cultures, and other cofactor(s) may be required for ISG20 to efficiently degrade the stem-loop region of HBV RNA. Our results thus have impacts on a better understanding of ISG20 biology and host-virus interaction, and may provide basis for host ribonuclease-based antiviral development.

## Results

### ISG20 basal expression in hepatocytes and induction by IFNs

**Fig 1:**
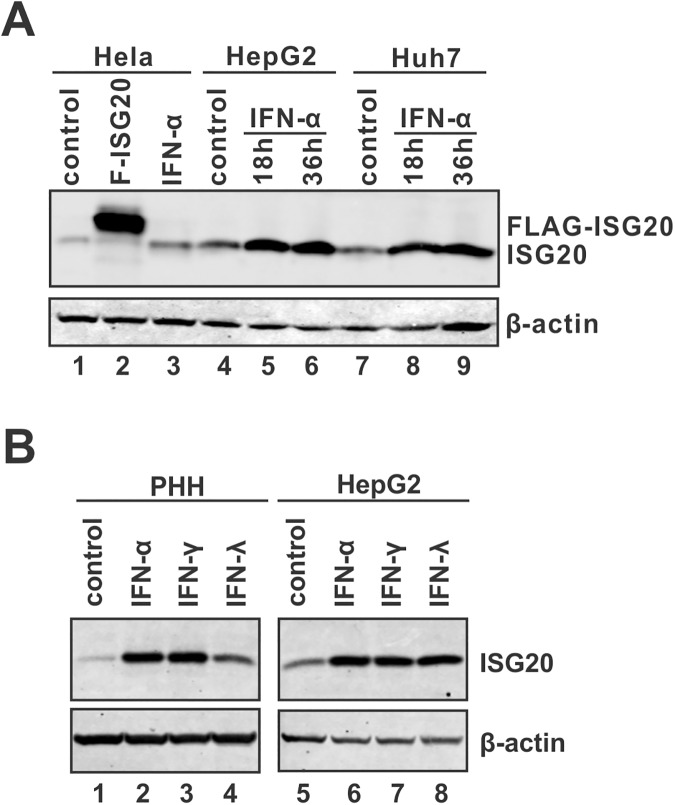
ISG20 expression in hepatocytes and its inducibility by IFNs. (A) Hela, HepG2, and Huh7 cells were seeded in a 12-well plate for overnight, then left untreated or treated with human IFN-α (1,000 IU/ml) for 18 or 36 h. The untreated cells (control) were harvested together with the cells treated by IFN-α for 36 h. ISG20 expression was detected by Western blot. Hela cells transfected with plasmid F-ISG20 expressing the FLAG-tagged ISG20 were used as positive control for ISG20 Western blot (lane 2). β-actin expression was presented as loading control. (B) PHHs and HepG2 cells were cultured in 12-well-plate and treated with type I IFN-α (1,000 IU/ml), type II IFN-γ (100 ng/ml) or type III IFN-λ (100 ng/ml), or left untreated (control) for 2 days. The levels of ISG20 expression were determined by Western blot.

ISG20 is an interferon inducible protein [16]. Firstly, we assessed the basal expression of ISG20 in cell cultures and its inducibility by IFN. Using Hela cells as a positive control, the basal level of ISG20 expression was detected in both HepG2 cells and Huh7 cells by Western blot, and its expression was significantly upregulated by IFN-α stimulation (Fig 1A). Furthermore, ISG20 can be highly induced by all three types of IFNs in both HepG2 cells and primary human hepatocytes (PHH) (Fig 1B). According to a previous study on the transcriptional regulation of ISG20, a unique interferon stimulated response element (ISRE) in the TATA-less human ISG20 promoter confers IRF-1-mediated constitutive transcriptional activity of ISG20 gene and responsiveness to both type I and II interferons [22]. The basal and cytokine-inducible expression of intrahepatic ISG20 indicates a potentially important role of ISG20 in host defense mechanisms against pathogen infections in hepatocytes.

### Overexpression of ISG20 inhibits HBV replication in cell cultures

**Fig 2:**
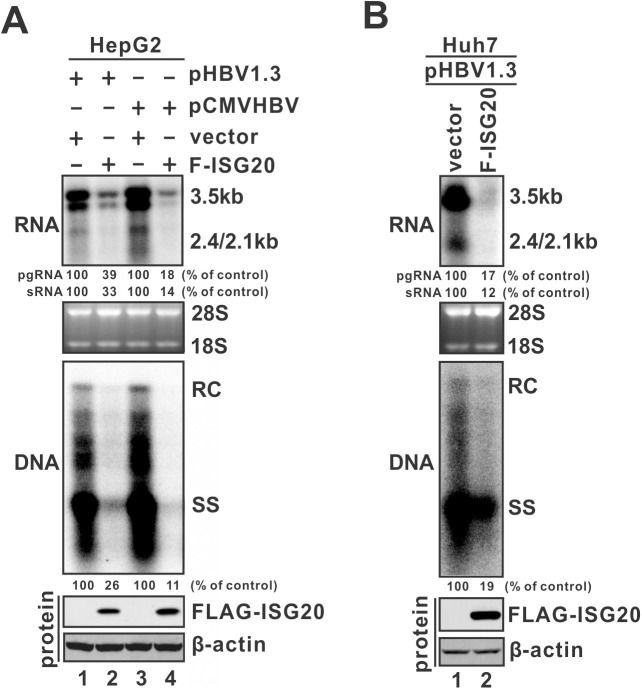
ISG20 overexpression reduces HBV replication in hepatocyte-derived cells. (A) HepG2 cells were co-transfected with either pHBV1.3 and F-ISG20 or empty vector, or pCMVHBV and F-ISG20 or empty vector, as indicated. Cells were harvested at day 5 post-transfection, and the levels of viral RNA and DNA were determined by Northern (top) and Southern (middle) blot hybridization, respectively. For RNA analysis, each lane was loaded with 10 μg of total RNA and probed with a genome-length, plus-strand-specific HBV riboprobe. Ribosomal RNAs (28S and 18S) are presented as loading controls. The positions of HBV pgRNA (3.5kb) and subgenomic surface RNAs (2.4kb and 2.1kb) are indicated. For DNA analysis, HBV core DNA was probed with genome-length, minus-strand-specific HBV riboprobe. The positions of relaxed circular (RC) and single-stranded (SS) DNAs are indicated. The relative pgRNA, sRNA or total DNA replicative intermediate level in each sample is expressed as the percentage of RNA or DNA of the cells transfected with empty vector. ISG20 overexpression was confirmed by Western blot using monoclonal antibodies against FLAG-tag. β-actin expression was presented as protein loading control (bottom panels). (B) The same experiment was done in Huh7 cells with pHBV1.3 as HBV expression vector.

In an effort to determine the antiviral activity of ISG20 against HBV replication in hepatocytes, FLAG-tagged wild type ISG20 was co-transfected with pHBV1.3 (HBV precore mRNA and pgRNA transcription is controlled by viral enhancer II and core promoter (EnII/Cp)) or pCMVHBV (pgRNA transcription is governed by CMV-IE promoter) in HepG2 cells. HBV viral RNA and DNA were analyzed, as shown in Fig 2A. The result clearly demonstrated that ISG20 markedly inhibited HBV DNA replication (middle panel) primarily through reducing the steady state levels of 3.5kb viral pgRNA (top panel) which is the template for HBV DNA synthesis, and such effect is independent of promoter (HBV EnII/Cp or CMV-IE) used to transcribe the pgRNA. A similar antiviral effect of ISG20 was also observed in Huh7 cells (Fig 2B). Furthermore, the ISG20-mediated pgRNA reduction is dose dependent (S1 Fig) and not due to a possible cytotoxicity effect caused by ISG20 overexpression (S2 Fig). In addition, along with pgRNA reduction, the levels of 2.4 kb and 2.1 kb HBV surface mRNA, which share 100% sequence identity with the 3’ portion of pgRNA, were also reduced upon ISG20 expression (Fig 2, top panels). The reduction of HBV 2.4/2.1 kb RNA by ISG20 was further confirmed by co-transfection of ISG20 and plasmid pLMS expressing HBV subgenomic surface mRNA in HepG2 cells (S3A Fig). Consistent with ISG20-mediated reduction of 3.5 kb and 2.4/2.1 kb HBV RNA, the levels of supernatant HBeAg and HBsAg were also significantly decreased (S3B Fig).

### ISG20 reduces HBV RNA by promoting RNA degradation

Considering that HBV plasmid DNA is the viral transcription template in the transfection system, it is possible that ISG20 reduces HBV RNA through destabilizing the transfected HBV plasmid DNA, or by blocking the nuclear import of input plasmid. To test these possibilities, we recovered the HBV plasmid DNA from the transfected cells by Hirt DNA extraction, followed by Dpn I digestion and Southern blot analysis. The result showed that the levels of input HBV plasmid DNA recovered from whole cells were equal in the absence or presence of ISG20, as revealed by Dpn I-digested fragments (S4A Fig, top panel). Furthermore, while ISG20 overexpression reduced HBV core DNA replication in cytoplasm, it did not affect the level of remaining cytoplasmic HBV plasmid DNA (S4A Fig, middle panel), further confirming that ISG20 does not alter the stability and distribution of the input HBV plasmid. In addition, ISG20 transfection in HBV stable cell line HepDES19 cells also resulted in the reduction of HBV RNA, which were transcribed from the integrated HBV DNA (S4B Fig). Collectively, these results clearly exclude any negative effect of ISG20 on the stability of HBV RNA transcription template, which is consistent with the reported inability of ISG20 to degrade double stranded DNA substrate *in vitro* [13].

Next, in order to determine whether ISG20-mediated down-regulation of HBV RNA *via* transcriptional or posttranscriptional mechanism, we first assessed the potential effect of ISG20 on viral promoter activity. As shown in S5 Fig, the luciferase reporter assays demonstrated that ISG20 did not significantly affect the activities of HBV Enhancer II/Core (EnII/Cp) promoter, S2 promoter, or CMV-IE promoter, but even enhanced HBV S1 promoter activity. Therefore, it is unlikely that ISG20 reduces HBV RNA through inhibiting viral promoter activity at the transcriptional level.

**Fig 3:**
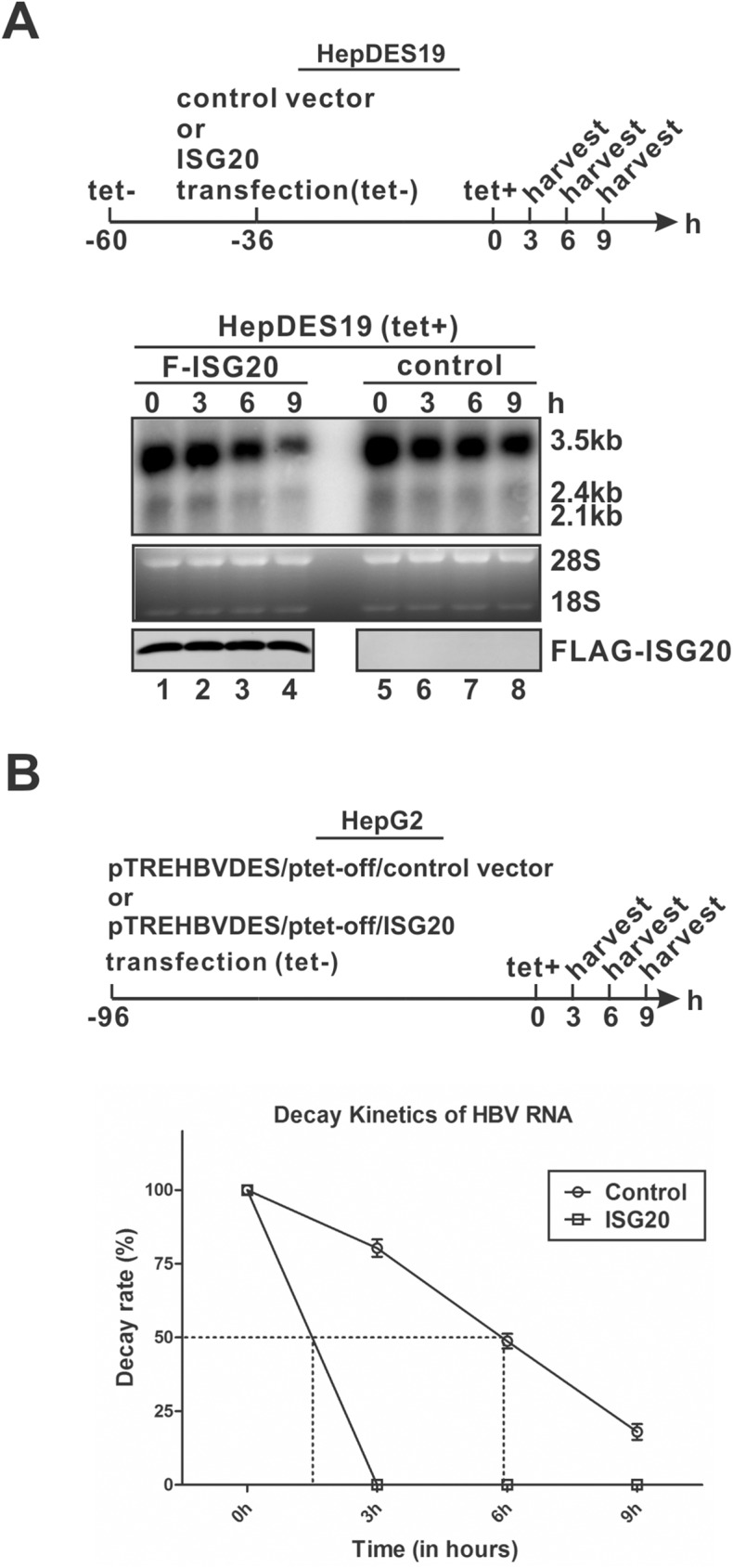
ISG20 promotes HBV RNA degradation in cell cultures. (A) HepDES19 cells were seeded in 35 mm-dish and cultured with tet-free medium to induce HBV pgRNA transcription. 24 h later, cells were transfected with 4 μg of control vector or plasmid F-ISG20 for 36 h, then tet was added back to the culture medium to shut down pgRNA transcription. Cells were harvested at indicated time points. HBV RNA was extracted from harvested samples and analyzed by Northern blot. Expression of FLAG-tagged ISG20 was detected by Western blot. The results are representative of three separate trials. (B) HepG2 cells in 12-well-plate were co-transfected with 0.7 μg of pTREHBVDES and 0.1 μg of pTet-off, plus 0.7 μg of control vector or plasmid F-ISG20. Four days post transfection, tet was added back and cells were harvested at indicated time points and subjected to HBV RNA qPCR analysis. The relative levels of HBV total RNA normalized to β-actin mRNA levels in each samples were expressed as the percentage of the RNA levels from the corresponding sample at 0 h time point (Mean ± SD, n = 4). The half-life of HBV RNA was marked on the plot.

We then compared the decay kinetics of HBV RNA in the absence and presence of ISG20 overexpression. Briefly, ISG20 expression vector or control empty vector was transfected into the inducible HBV stable cell line HepDES19 cells in the absence of tetracycline (tet) to induce HBV pgRNA transcription and ISG20 expression; 36 h later, tet was added back to shut down the *de novo* pgRNA transcription from the transgene, and after that, the levels of HBV RNA at different time points were analyzed by Northern blot (Fig 3A). The representative Northern blot demonstrated that the levels of HBV RNA in HepDES19 cells overexpressing ISG20 at each time point were less than that in the control group, suggesting that ISG20 promotes HBV RNA degradation. This is consistent with the previous observations that ISG20 functions as the 3’-5’ riboexonuclease to degrade single-stranded viral RNA genomes [14, 16]. It is worth noting that not every HBV RNA-containing HepDES19 cells were transfected by ISG20, therefore, the actual effect of ISG20 on HBV RNA stability ought to be stronger than observed in this experiment. To further quantitatively validate the observed enhancement of HBV RNA degradation by ISG20, we transfected HepG2 cells with tetracycline-inducible HBV expression vector pTREHBVDES and plasmid pTet-off expressing the tetracycline-responsive transcriptional activator (tTA), together with either control vector or ISG20 plasmid. In this way, both HBV RNA and ISG20 coexisted in same cells. Four days later, similarly to the above experiment in HepDES19 cells, tetracycline was added back to block new round of pgRNA transcription, and the time course HBV RNA level was quantified by qPCR. As shown in Fig 3B, the regular half-life of intracellular HBV RNA was approximately 6 h, which is consistent with previous measurements [10, 23], but the decay kinetics of HBV RNA was much faster in cells transfected with ISG20, of which the HBV RNA half-life was much less than 3 h, suggesting that ISG20 promotes HBV RNA decay. Hence, we conclude that ISG20-mediated HBV RNA reduction is primarily through downregulation of HBV RNA stability.

### Antiviral effect of endogenous ISG20 on HBV infection

**Fig 4:**
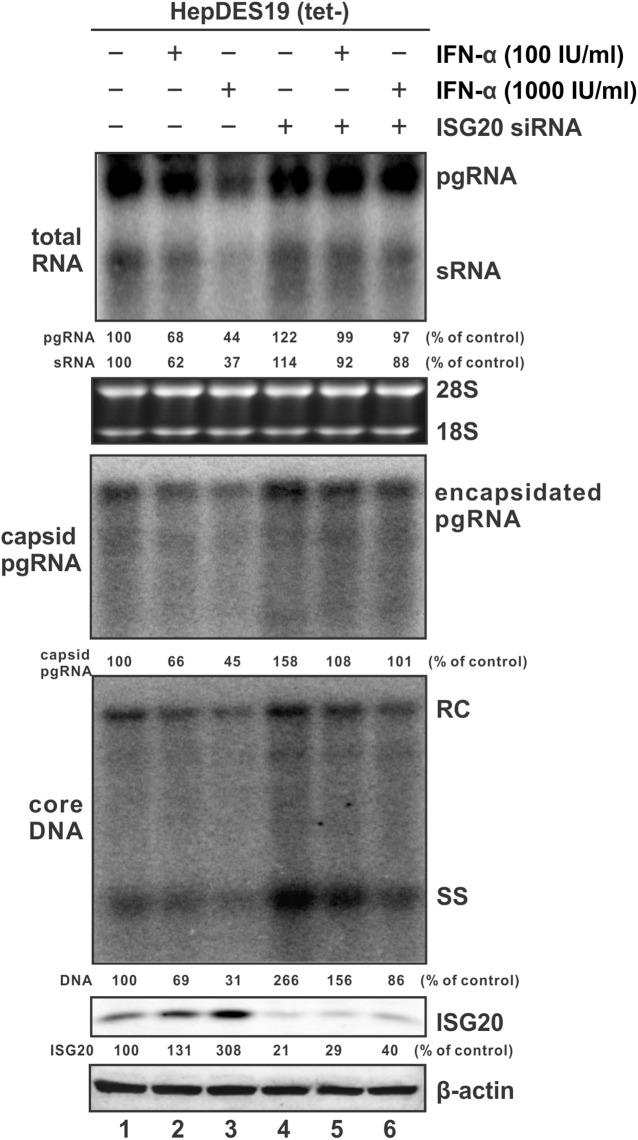
The antiviral effect of ISG20 in HepDES19 cells under basal expression and IFN-α treatment. HepDES19 cells were transfected with 100 nM of control siRNA (Lane 1–3) or ISG20 siRNA (siISG20) (lane 4–6) twice with a 24 h interval after tet being withdrawn. Culture medium was replaced 12 h after the 2nd siRNA transfection, and cells were either left untreated as controls (lane 1 &4) or treated with 100 IU/ml (lanes 2 & 5) or 1,000 IU/ml (lanes 3 & 6) of IFN-α. Cells were harvested 5 days after 2nd transfection. Viral total RNA (top panel), encapsidated pgRNA (upper middle panel), and core DNA (lower middle panel) were subjected to Northern and Southern analyses, respectively. ISG20 protein expression was revealed by Western blot, and β-actin served as loading control (bottom panels). The relative levels of viral nucleic acids and ISG20 expression in the siISG20 transfected or IFN-α treated samples (lanes 2–6) are expressed as the percentage of the control sample (lane 1). The data presented here are representative of two independent experiments.

There is a basal level of ISG20 expression in hepatocytes and that could be further induced by interferons (Fig 1). It was therefore of interest to assess the antiviral activity of endogenous ISG20 against HBV infection under its basal and induced levels. Firstly, we performed the test in HBV stable cell line HepDES19 [24]. As shown in Fig 4, knock down of ISG20 expression in the absence of IFN-α treatment resulted in a marked upregulation of the steady state levels of HBV total RNA, encapsidated pgRNA, and DNA (comparing lane 4 to lane l). The observed relatively greater upregulation of core DNA than viral RNA upon knock down of ISG20 might be due to the longer lifespan of DNA. When the cells were treated with IFN-α, ISG20 was upregulated, and viral RNA and DNA were reduced, in an IFN dose-dependent manner (lanes 1–3). It is worth to note that the IFN-elicited reduction of HBV RNA in HepDES19 cells was less profound than in HBV-transfected cells with ISG20 overexpression (compared Fig 4 to Fig 2). Such difference might be due to the reported weak response of hepatoma cells to IFN treatment [25], and/or the potential unknown negative regulator(s) of ISG20 co-induced by IFN. Nonetheless, in the cells treated with IFN-α and ISG20 siRNA, the levels of HBV RNA were restored to the untreated control level (comparing lanes 5 and 6 to lanes 1–3), suggesting that ISG20 plays a major role in IFN-mediated reduction of HBV RNA in HepDES19 cells. However, neither viral RNA nor DNA reached their levels in cells treated with ISG20 siRNA only (comparing lanes 5 and 6 to lane 4), indicating that other ISGs are involved in IFN-mediated suppression of HBV DNA replication at multiple steps.

**Fig 5:**
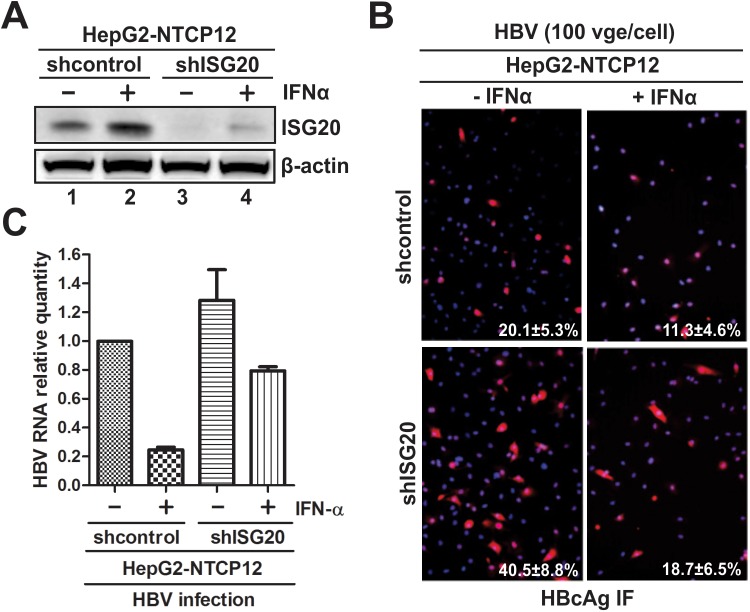
The antiviral effect of ISG20 in HBV infection system. HepG2-NTCP12 cells stably transduced by control lentiviral shRNA (shcontrol) or ISG20 lentiviral shRNA (shISG20) were spinoculated with HBV at 100 vge/cell. 16 h later, the infected cells were mock treated or treated with 1,000 IU/ml of IFN-α for 6 days, and the cells were subjected to the following analyses: (A) The expression of ISG20 was analyzed by Western blot. (B) HBV infectivity was assessed by HBcAg immunofluorescence, and the percentage of HBcAg-positive cells were calculated from multiple microscopic field of view (mean±SD, n = 5). Nuclei were stained with DAPI. (C) HBV total RNA were quantified by qPCR and the relative expression levels to β-actin mRNA were plotted as fold change to control samples (HBV infected shcontrol cells without IFN-α treatment) (mean±SD, n = 3).

Next, we assessed the antiviral function of ISG20 in an HBV *in vitro* infection system, specifically, the viral receptor NTCP reconstituted HepG2 cells [26]. The endogenous ISG20 in HepG2-NTCP12 cells was stably inhibited through lentiviral shRNA transduction. The obtained ISG20 knock down cell line and control knock down cell line were then infected with HBV and treated with or without IFN-α. As shown in Fig 5A, the basal ISG20 expression was successfully knocked down in HepG2-NTCP12-shISG20 cells (comparing lane 3 to lane1), and IFN-α significantly upregulated ISG20 expression in the HepG2-NTCP12-shcontrol cells (comparing lane 2 to lane1), but only slightly induced ISG20 in the HepG2-NTCP12-shISG20 cells (comparing lane 4 to lane3). Then the outcomes of HBV infection were analyzed. The HBcAg immunofluorescence staining demonstrated that knock down of ISG20 resulted in a higher percentage of HBV positive cells when compared to control cells without or with IFN-α treatment (Fig 5B). Because that the ISG20-mediated HBV RNA degradation will lead to an overall suppression of the entire HBV life cycle in the infection system, we infer that the increased percentage of core staining-positive cell number in HBV infected HepG2-NTCP-shISG20 cells is a combinational end effect of increased viral protein expression, DNA replication, and cccDNA synthesis, plus the possible higher virus spread rate. Since HBV RNA is a more convincing marker for HBV infection and the authentic antiviral target of ISG20, the qPCR assay of HBV total RNA from the infected cells further demonstrated that IFN-α is able to reduce HBV RNA in infected cells, and knocking down of ISG20 greatly favors HBV infection in both IFN-treated and untreated cells (Fig 5C). Consistent with the observations in HepDES19 cells (Fig 4), inhibition of ISG20 significantly but partially abrogated the overall antiviral activity of IFN-α in HBV infected cells (Fig 5B and 5C), presumably due to the induction of other antiviral ISGs. Collectively, the above data suggested that ISG20 serves as a host intrinsic restriction factor to limit HBV infection under both basal expression and cytokine induction.

### ISG20-mediated HBV RNA decay depends on its ribonuclease activity

**Fig 6:**
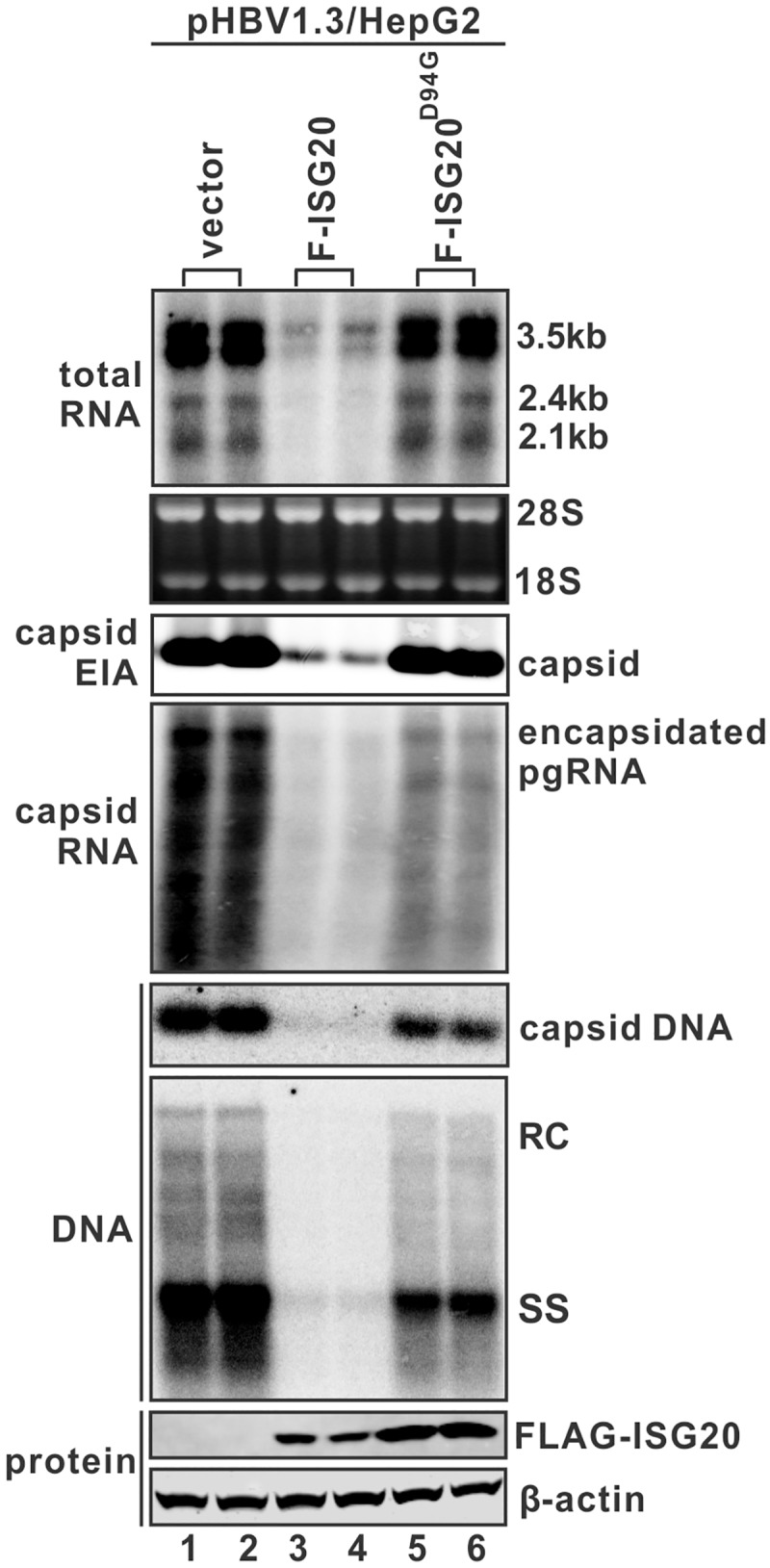
The enzymatically inactive form of ISG20 (ISG20^D94G^) is defective to degrade HBV RNA but retains antiviral activity against pgRNA encapsidation. HepG2 cells were transfected with pHBV1.3 and equal amount of control vector (lanes 1 & 2) or F-ISG20 (lanes 3 & 4) or F-ISG20^D94G^ (lanes 5 & 6). Cells were harvested 5 days post-transfection and levels of HBV RNA (1st panel from the top) and encapsidated pgRNA (4th panel from the top) were determined by Northern blot hybridization. The assembled HBV capsid was revealed by native capsid gel EIA assay (3rd panel from the top) and the viral DNA in capsid was detected *in situ* by hybridization (5th panel from the top). HBV core DNA replicative intermediates were extracted and analyzed by Southern blot (6th panel from the top). Expression of FLAG-tagged ISG20 proteins was revealed by Western blot and β-actin served as loading control (bottom two panels). Results from duplicate experiments are presented.

ISG20 has three putative exonuclease domains and it has been reported that a single D94G mutation in the Exo II domain completely abolished the protein’s enzymatic activity *in vitro* [13]. In line with this, we found that the enzymatic inactive mutant F-ISG20^D94G^ failed to reduce HBV RNA in HepG2 cells (Fig 6, top panel), suggesting that ISG20-mediated HBV RNA decay requires its exoribonuclease activity. Interestingly, although the levels of total viral RNA and capsid remained the same, the level of encapsidated HBV pgRNA was significantly reduced under the expression of ISG20^D94G^ mutant (middle panel, comparing lanes 5&6 to 1&2), which consequently led to the inhibition of viral DNA replication (bottom panels). The inhibitory effect of ISG20^D94G^ on pgRNA encapsidation was further confirmed with HBV genome harboring polymerase Y63D mutation, which supports HBV pgRNA binding and encapsidation but not DNA replication (S6 Fig)

Since HBV pgRNA encapsidation requires the dynamic interactions among three viral components, specifically the pgRNA, Pol, and core protein [27], it is possible that ISG20^D94G^ might be antagonizing one or more of these three viral factors to block encapsidation. However, ISG20^D94G^ did not inhibit capsid assembly (Fig 6, 3rd panel from the top), and ISG20-mediated HBV RNA degradation does not rely on viral core or pol protein (S7 Fig). Based on these data, we hypothesized that ISG20 might physically bind to HBV RNA for degradation, and thus in the particular case of ISG20^D94G^, its association with pgRNA inhibited the binding of HBV polymerase to pgRNA and the subsequent pgRNA encapsidation.

### ISG20^D94G^ interacts with HBV RNA and competes Pol-pgRNA binding in cell cultures

**Fig 7:**
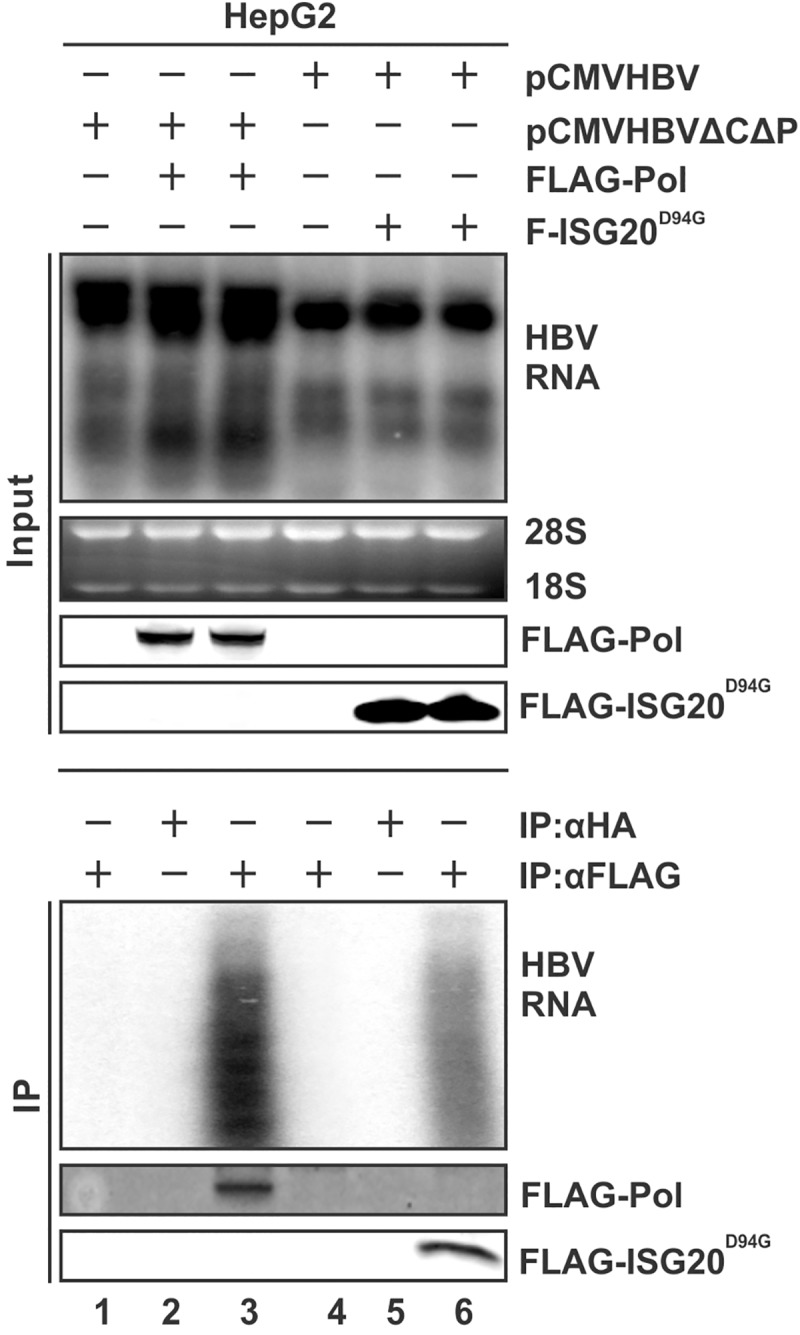
ISG20^D94G^ physically associates with HBV RNA in cells. HepG2 cells were co-transfected with plasmid pCMVHBVΔCΔP and either control vector (lane 1) or FLAG-Pol (lanes 2&3), or pCMVHBV with either control vector (lane 4) or F-ISG20^D94G^ (lanes 5&6). Cells were harvested 4 days post-transfection. Input HBV RNA was determined by Northern blot (top panels). Input FLAG-Pol and F-ISG20^D94G^ proteins were determined by Western blot using FLAG Ab (top panels). Cell lysates were immunoprecipitated with beads coated with FLAG Ab, the immunoprecipitated Pol and ISG20^D94G^ were revealed by Western blot using FLAG Ab (lanes 3&6, bottom panel), and the bound RNA was extracted by Trizol and analyzed by Northern blot (lanes 3&6, bottom panel). HA Ab pull-down served as negative controls (lanes 2 & 5, bottom panel). See Material and Methods for more experimental details.

To test the above hypothesis, we first investigated the association between ISG20 and HBV RNA in cell culture. In this immunoprecipitation (IP)-Northern assay (Fig 7), plasmid pCMVHBVΔCΔP which supports HBV RNA transcription but does not support pgRNA encapsidation *in cis* due to the deletion of core and Pol expression was used as HBV RNA transcription template, and FLAG-tagged HBV Pol (FLAG-Pol) was used *in trans* as positive control for pgRNA binding. To avoid the rapid RNA degradation mediated by wild type ISG20 expression, ISG20^D94G^ mutant was used for this assay. The upper panel of Fig 7 indicates the input HBV RNA level and FLAG-Pol or F-ISG20^D94G^ protein levels. The lower panel of Fig 7 shows the Northern blot results after IP with FLAG antibody (Ab). As expected, FLAG-Pol interacted with HBV RNA, as FLAG Ab, but not the HA Ab, could pull down the HBV RNA. Same as FLAG-Pol, ISG20^D94G^ also specifically formed complex with HBV RNA in this IP-Northern assay, suggesting that ISG20 interacts with HBV RNA in cell cultures, though it remains unknown whether the interaction is direct or indirect.

**Fig 8:**
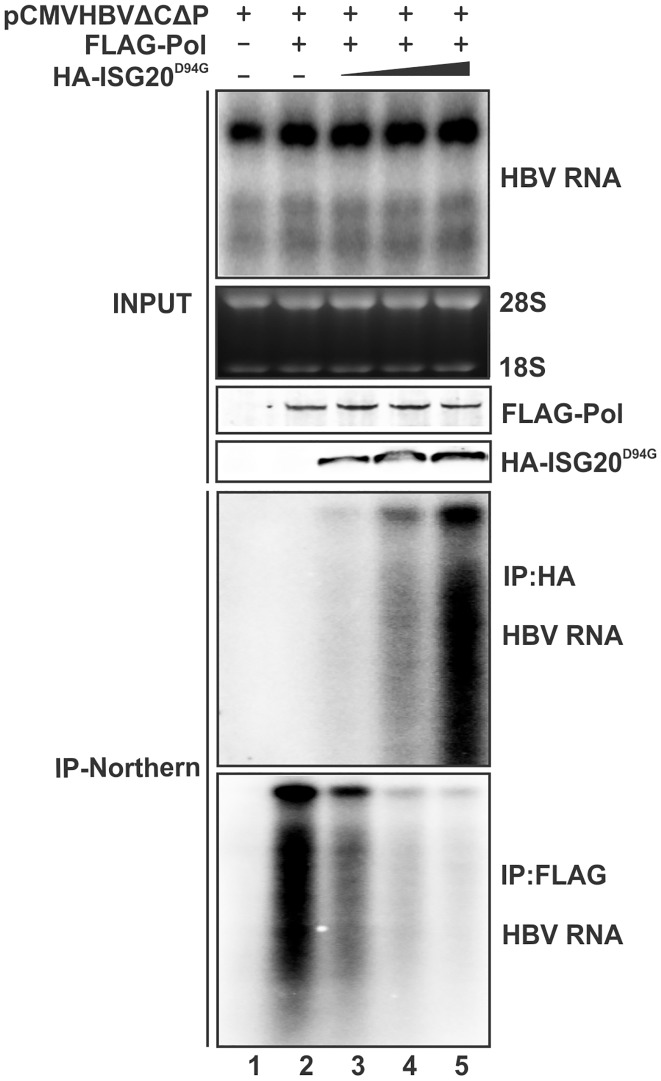
ISG20^D94G^ competes HBV Pol for binding to pgRNA in cell cultures. HepG2 cells in 6-well-plate were co-transfected with 1 μg of pCMVHBVΔCΔP and 5 μg control vector (lane 1) or 1 μg of FLAG-Pol in the absence of HA-ISG20^D94G^ (lane 2) or increased amount of HA-ISG20^D94G^ (1 μg, 2 μg, 4 μg; lanes 3–5). The total amount of transfected DNA was kept constant (6 μg/well) by adding control vector plasmid (lanes 2–4). 5 days later, total cellular HBV RNA and protein (FLAG-Pol and HA-ISG20^D94G^) were determined by Northern and Western blot, respectively, as input controls (top panels). Immunoprecipitation was performed by using antibodies against HA or FLAG epitopes, followed by Northern blot analysis of HBV RNA (bottom panels).

Next, we set out to investigate the potential competitive effect of ISG20 on Pol-pgRNA binding. Fixed amount of FLAG-Pol and pCMVHBVΔCΔP were co-transfected into cells together with plasmid expressing HA-tagged ISG20^D94G^ (HA-ISG20^D94G^) in a titrated manner. Fig 8 shows the input HBV RNA and FLAG-Pol and HA-ISG20^D94G^ protein levels (upper panels) and the immunoprecipitated HBV RNA levels after HA or FLAG Ab pull-down (the lower panels). The HA Ab IP-Northern result demonstrated that ISG20^D94G^ bound to HBV RNA in a dose dependent manner. In contrast, increased amount of ISG20^D94G^ resulted in the decreased amount of HBV RNA associated with Pol, suggesting that ISG20^D94G^ competes with HBV Pol for binding of pgRNA and thus inhibits HBV pgRNA encapsidation. It is assumed that the wildtype ISG20 may possess additional antiviral function as what ISG20^D94G^ does in cells, but the inhibition of pgRNA encapsidation, if any, will likely be overwhelmed by its dominant primary effect on RNA degradation.

### Mapping the ISG20 responsive elements in HBV RNA genome

**Fig 9:**
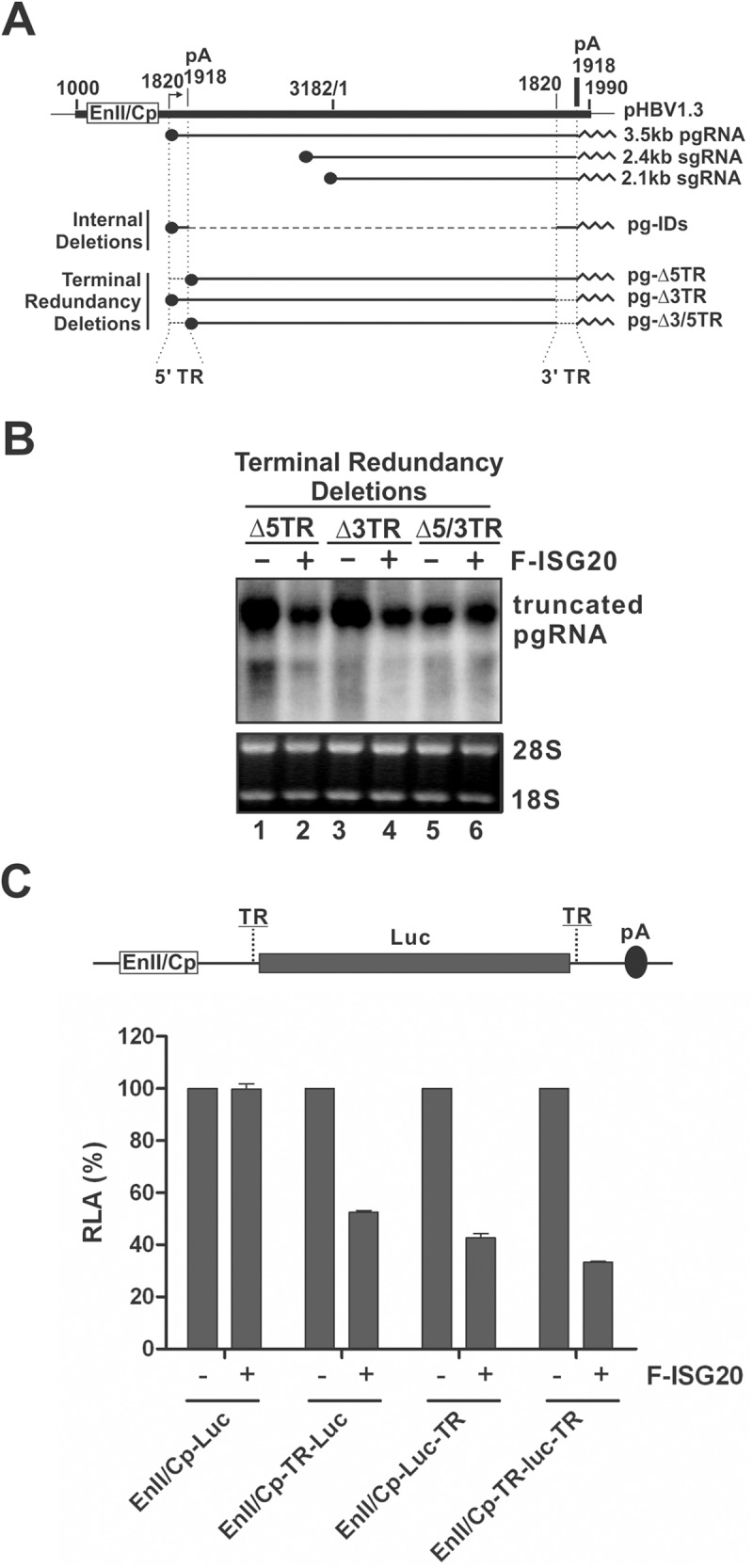
Mapping the ISG20 responsive elements in HBV genome. (A) Schematic illustration of HBV pgRNA deletion clones. Plasmid pHBV1.3 contains a 1.3 overlength HBV genome (Genbank Accession Number U95551), starting at nt 1000. The HBV nucleotide positions are according to Galibert et al [28]. Cp represents the HBV core promoter. pA is the polyadenylation site. The arrow indicates the pgRNA transcription initiation site (nt 1820). Three major HBV mRNA (3.5 kb, 2.4 kb, and 2.1 kb) are depicted underneath the 1.3 mer HBV DNA template. The solid dot indicates 5’ cap of mRNA; and the sawtooth line represents the polyA tail at the 3’ terminus of mRNA. The internal deletion clones (pg-IDs) are described in details in S8 Fig. The terminal redundancy (TR) deletion clones contain truncations of HBV sequences (nt 1820–1918) at either 5’ or 3’ terminus of pgRNA coding sequences (pg-Δ5TR and pg-Δ3TR, respectively.), or both (pg-Δ5/3TR). The transcription of terminal truncated pgRNA is governed by CMV-IE promoter in the pCDNA3.1/V5-His-TOPO vector. (B) Sensitivity of HBV RNA with TR deletion to ISG20-mediated RNA reduction. HepG2 cells were transfected with HBV TR deletion clone and control plasmid or F-ISG20 plasmid. Cells were harvested at day 4 post transfection and subjected to viral RNA analysis by Northern hybridization. **(**C) HBV TR insertion renders Luc gene to be sensitive to ISG20. The schematic illustration indicates the reporter construct EnII/Cp-Luc with HBV TR insertion at the flanking non-translational region of luciferase ORF. HepG2 cells were transfected with each indicated reporter plasmid and control vector or plasmid expressing ISG20. Cells were lysed at day 3 post transfection and luciferase activity was measured. The plotted relative luciferase activity (RLA) represents the mean ± SD (n = 3) of the percentage of absorbance obtained from wells transfected with ISG20 over control vector.

The above data demonstrated that ISG20 interacts with HBV RNA. In order to map the ISG20-targeting sequences, we made use of a series of internal deletion clones and terminal deletion clones of the 3.5kb HBV RNA genome to express truncated RNAs and their sensitivities to ISG20-mediated RNA degradation were assessed (Fig 9A and S8A Fig). Firstly, we found that all those pgRNA mutants with internal deletions (within nt 2009-3182/1-1574) were sensitive to ISG20 (S8B Fig). Then the terminal redundancy (TR, nt 1820–1918, containing ε (nt 1849–1909)) mutants with deletion of either 5’ TR (Δ5TR) or 3’ TR (Δ3TR) from pgRNA were tested. Interestingly, while the single TR deleting pgRNA remained sensitive to ISG20 (Fig 9B, lanes 1–4), the pgRNA mutant with both 5’ and 3’ TR removed (Δ5/3TR) became completely resistant (Fig 9B, lanes 5–6), indicating that one copy of the TR sequences is sufficient to confer the susceptibility of HBV pgRNA to ISG20-mediated RNA degradation. This is also consistent with the fact that ISG20 is also able to degrade HBV subgenomic RNAs which only contain the 3’ TR (Fig 2 and S3 Fig).

To further confirm that the HBV TR is the ISG20 responsive element, HBV core promoter activity reporter plasmids EnII/Cp-Luc with TR region inserted at the 5’ (EnII/Cp-TR-Luc), or 3’ (EnII/Cp-Luc-TR), or both 5’ and 3’ (EnII/Cp-TR-Luc-TR) non-translational region of luciferase gene (Fig 9C) were transfected into HepG2 cells with or without F-ISG20. The luciferase activities were measured and the result demonstrated that the introduction of HBV TR into luciferase mRNA resulted in a significant reduction of luciferase gene expression under ISG20 expression (Fig 9C). Therefore, we conclude that ISG20 selectively targets TR sequences of HBV RNA to initiate RNA degradation.

### ISG20 directly binds to epsilon of HBV TR sequence *in vitro*

**Fig 10:**
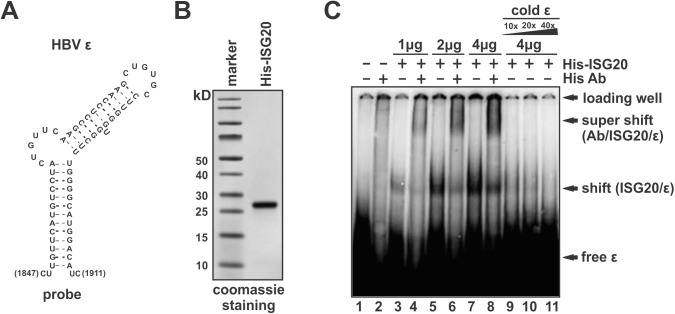
ISG20 binds to HBV ε *in vitro*. (A) Schematic stem-loop structure of HBV ε RNA. Ribonucleotide sequences (nt 1847–1991, genotype D, subtype ayw) are presented with base paring indicated by dotted line. (B) Verification of the purified recombinant 6×His-tagged ISG20 by SDS-PAGE Coomassie staining. (C) EMSA assay of ISG20-ε binding. The indicated amount of ISG20 proteins were incubated with 100 ng ^32^P-end-labeled ε RNA in binding buffer to form nucleoprotein complexes. Monoclonal anti-His antibody was used for supershifting of the His-ISG20/ HBV ε complex. Excessive amount of cold unlabeled HBV ε RNA (10×, 20×, 40×) were used to compete with the binding of ISG20 to 100 ng radiolabeled HBV ε. The nucleoprotein complexes were separated by native PAGE and the shifted bands were detected by autoradiography.

HBV Pol recognizes and binds on a stem-loop structure in 5’ TR region of HBV pgRNA, called epsilon (ε), to initiate pgRNA encapsidation and reverse transcription [29]. Since ISG20-mediated HBV RNA degradation relies on TR sequence (Fig 9) and ISG20^D94G^ competes with Pol for HBV RNA binding (Fig 8), it is therefore of immediate interest to investigate whether ISG20 also directly binds on HBV ε to exert its antiviral functions. To this end, an electrophoretic mobility shift assay (EMSA) was employed. As shown in Fig 10, purified recombinant His-tagged ISG20 proteins were incubated with 5’ radiolabeled HBV ε RNA *in vitro* and the protein/RNA complexes were analyzed *via* the mobility shifting of the RNA in the native polyacrylamide gel. The proper folding of stem-loop structure of ε RNA was confirmed by recombinant Pol/ε complex formation as detected in the EMSA assay (S9 Fig). Interestingly, in the absence of other cellular proteins, His-ISG20 could directly, dose-dependently bind on ε RNA, shown as the shift bands in Fig 10C (lanes 3, 5, 7), and the binding specificity between His-ISG20 and ε was confirmed by the His-antibody(Ab) /His-ISG20/ε super-complex formation detected as a super-shift in EMSA (lanes 4, 6, 8). Competition of ISG20 binding to the radiolabeled ε RNA by excess amount of cold ε probes indicated again that ISG20 specifically binds to HBV ε (lanes 9, 10, 11). It is of note that the incubation of ISG20 and ε RNA during EMSA was performed in protein/RNA binding buffer without manganese, an essential ion for the enzymatic activity of ISG20 [13], therefore the degradation of ε RNA, if any could occur, would not be observed in this binding assay.

**Fig 11:**
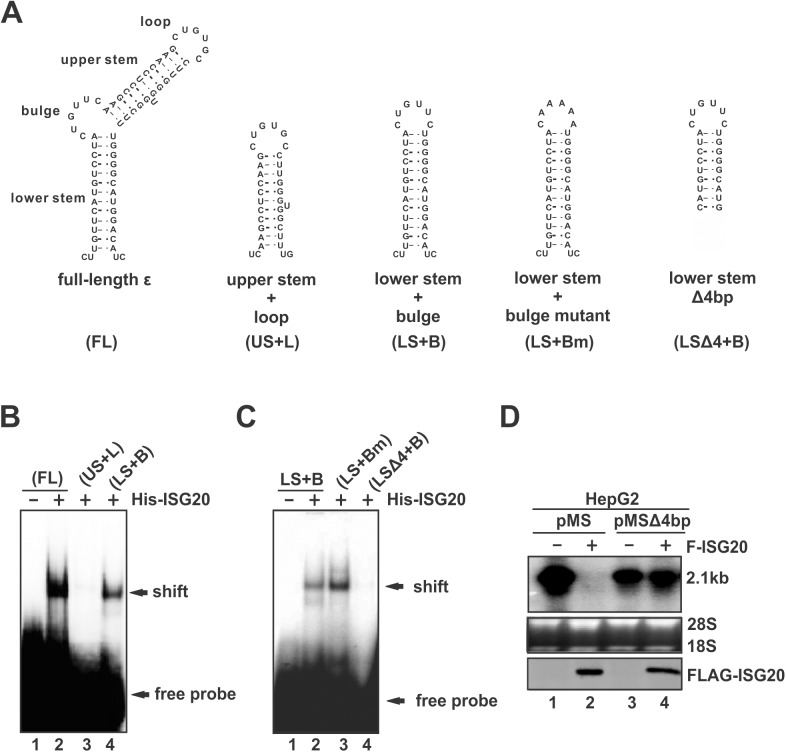
ISG20 specifically binds to lower stem of HBV ε. (A) Schematic illustrations of the wildtype HBV ε RNA and mutants. The substructural domains of ε, including the lower stem, bulge, upper stem, and apical loop, are marked on the full-length form. Shorter versions of ε include the upper stem loop (US+L), lower stem with wildtype or mutant bulge sequence as loop (LS+B, LS+Bm), and LS+B with bottom 4 base-pairs removed from the lower stem (LSΔ4+B). These RNA fragments were chemically synthesized and 5’ end radiolabeled for ISG20 EMSA. (B) EMSA of ISG20 binding with full-length (FL) ε, US+L, and LS+B. (C) EMSA of ISG20 binding with LS+B, LS+Bm, and LS+B. (D) HepG2 cells were transfected with plasmid pMS transcribing the 2.1kb HBV RNA, or pMSΔ4bp transcribing the 2.1kb HBV with 4 nucleotides removed from the bottom right arm of the lower stem of ε, in the absence or presence of F-ISG20. HBV RNA and FLAG-tagged ISG20 were analyzed by Northern and Western blot, respectively.

To further dissect the substructural domain of ε targeted by ISG20, the synthetic upper stem-loop RNA and the lower stem-loop RNA of ε and their mutants, as shown in the Fig 11A, were radiolabeled and used in gel shift assay. Interestingly, ISG20 bound to the lower stem with bulge sequence serving as loop, but not the upper stem-loop of ε in EMSA (Fig 11B), which indicates that ISG20 recognizes the lower stem and/or bulge of HBV ε as its binding site. To further determine the binding position within the lower stem and bulge region, the bulge sequences were mutated or the lower stem were truncated from the bottom (Fig 11A), and used as probes in EMSA. Interestingly, we found that the loop (or bulge) sequence is not critical for ISG20 binding as long as the lower stem is intact; however the bottom 4 base-pairs of the stem is indispensable for ISG20 binding (Fig 11C). To further confirm the requirement of these 4 base-pairs for ISG20 to carry out its biological functions, we disrupted the base pairing at the bottom of ε stem-loop in HBV 2.1 kb surface mRNA by removing 4 nucleotides from the 3’ end of ε coding sequence, and found that the mutant RNA became resistant to ISG20-mediated RNA degradation in cell cultures (Fig 11D). Collectively, these findings imply that ISG20 recognizes sequence-specific secondary structure of HBV ε for RNA binding and degradation.

### Exo III domain mediates the binding of ISG20 to ε

**Fig 12:**
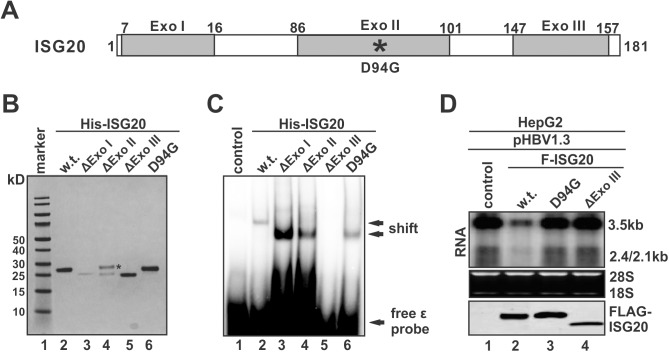
Exo III motif is responsible for ISG20 to bind ε. (A) Schematic illustration of ISG20. The amino acid (a.a) positions are labeled with numbers. The gray boxes indicate the predicted Exo motifs. The enzymatic mutant site (D94G) is marked with an asterisk. (B) Bacterially expressed His-tagged ISG20 and mutants were purified and examined by SDS-PAGE Coomassie staining. The asterisk indicates a nonspecific protein band co-purified with the recombinant ΔExoII mutant. (C) EMSA of ε binding by wildtype ISG20 and the indicated mutants. (D) HepG2 cells were co-transfected with pHBV1.3 and control vector or indicated FLAG-ISG20 constructs. HBV RNA and ISG20 proteins were detected by Northern and Western blot, respectively.

Since ISG20 is able to bind on its RNA substrate directly in the absence of any other cellular or viral proteins, it is therefore of interest to investigate which domain of ISG20 is responsible for RNA binding. Three distinct exonuclease motifs, namely Exo I (a.a7-16), Exo II (a.a 86–101) and Exo III (a.a 147–157), have been predicted in ISG20 protein sequence from previous studies [14, 30] (Fig 12A). Based on that, the recombinant mutant ISG20 proteins with each Exo domain deletion or Exo II D94G mutation were tested for ε binding activity by EMSA. As shown in Fig 12B and 12C, in comparison to wildtype ISG20, deletion of Exo I or Exo II did not reduce, but even increased the binding affinity of ISG20 with ε (lanes 2–4); however ISG20 with Exo III deletion completely lost the binding activity (lane 5), indicating that the Exo III is the functional binding domain of ISG20 for its RNA substrate. In addition, the enzymatically inactive D94G mutant retains its function for ε binding (lane 6), which is consistent with the observed association of ISG20^D94G^ with HBV RNA and subsequent inhibition of pgRNA encapsidation in cells (Figs 6–8). It is worth noting that the ΔExo I and ΔExo II deletion mutants, as well as the ISG20^D94G^ mutant, exhibited altered mobility compared to wildtype ISG20 in the gel shift assay (Fig 12C, lane 2 vs. lanes 3, 4, 6). While it is predictable that the faster mobility of ΔExo I:ε and ΔExo II:ε complexes is likely due to the protein’s lower molecular weight (MW), the lower shift of ISG20^D94G^:ε complex is probably because that such RNP complex exhibits a different structural conformation compared to the ISG20:ε complex.

To confirm the function of Exo III domain for ISG20 activity, the FLAG-tagged ISG20 protein with a deletion of Exo III domain was expressed in HBV transfected cells. As shown in Fig 12D, while the wildtype ISG20 significantly reduced HBV RNA levels as expected, the ISG20ΔExoIII mutant was similar to ISG20^D94G^ in inefficiency in reduction of HBV RNA. Therefore, we conclude that, while ISG20 requires Exo II domain for its enzymatic activity, the separate Exo III domain is responsible for RNA substrate binding of ISG20, which is a prerequisite for RNA degradation.

### Cofactor(s) is required for ISG20 to efficiently degrade ε RNA

**Fig 13:**
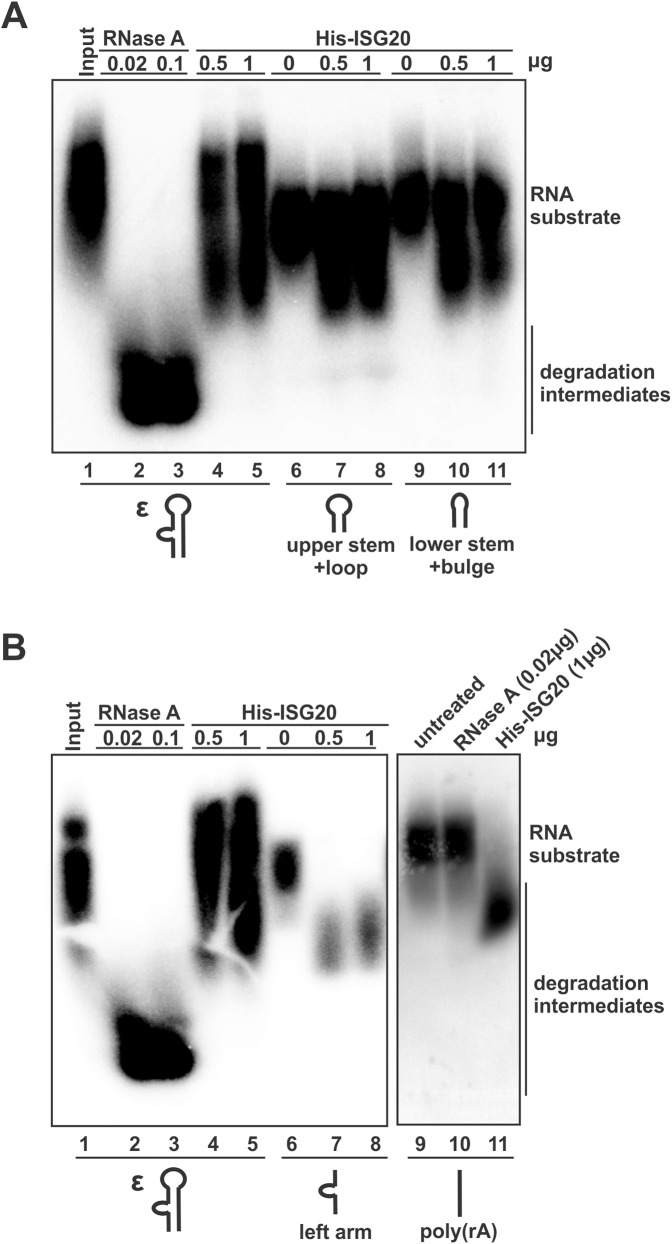
ISG20 requires cofactor(s) to efficiently degrade ε RNA. 0.1 µg of 5’-radiolabeled synthetic RNA substrates, specifically (A) the intact ε, upper stem-loop region (US+L), and lower stem with bulge serving as loop (LS+B); and (B) the intact ε, single-stranded left arm portion of ε, and 30-mer poly(rA), were incubated with the indicated amount of RNase A or purified His-ISG20 in nuclease reaction buffer for 15 min, then the reactions were terminated and the mixtures were fractionated through 10% TBE-Urea denaturing polyacrylamide gel, and the dried gel was subjected to autoradiography.

Lastly, we set out to characterize the enzymatic activity of ISG20 against ε RNA *in vitro*. The previous biochemistry study has demonstrated that ISG20 catalyzes RNA degradation *in vitro* in a manganese-dependent manner [13]. As shown in Fig 13, under the reaction condition that supports ISG20 ribonuclease activity [13], a wide spectrum endoribonuclease RNase A efficiently degraded ε RNA as a positive control (panel A and B, lanes 1–3). However, ISG20 exhibited much less efficiency to degrade the full length ε RNA substrate (panel A and B, lanes 1, 4–5), and other two stem-loop subdomains (US+L, LS+B) (panel A, lanes 6–11), suggesting that ISG20 alone does not favor RNA with secondary structures for degradation. Such observation is consistent with a previous study showing that ISG20 operated poorly on double-stranded regions of RNA substrate *in vitro* [13]. In line with these, we found that ISG20 was able to catalyze the degradation of RNA substrate derived from the left arm of ε (panel B, lanes 6–8), which is lack of secondary structures. Furthermore, ISG20 efficiently degraded the single-stranded linear 30-mer poly(rA) oligo *in vitro* (panel B, lane 11), confirming that the recombinant ISG20 protein used in the assay was enzymatically active. As a control, RNase A was unable to degrade poly(rA) because it only cleaves RNA string at the 3´ end of pyrimidine (rC or U) residues (panel B, lane 10) [31]. Since the RNA substrates were 5’ radiolabeled, the pattern of degradation intermediates generated by ISG20 is consistent with the 3’-5’ exoribonuclease activity of ISG20 (panel B, lanes 7, 8, 11). Collectively, the above *in vitro* data indicate that other host cofactor(s) may be required to coordinate with ISG20 for the observed efficient HBV RNA degradation in cells.

## Discussion

**Fig 14:**
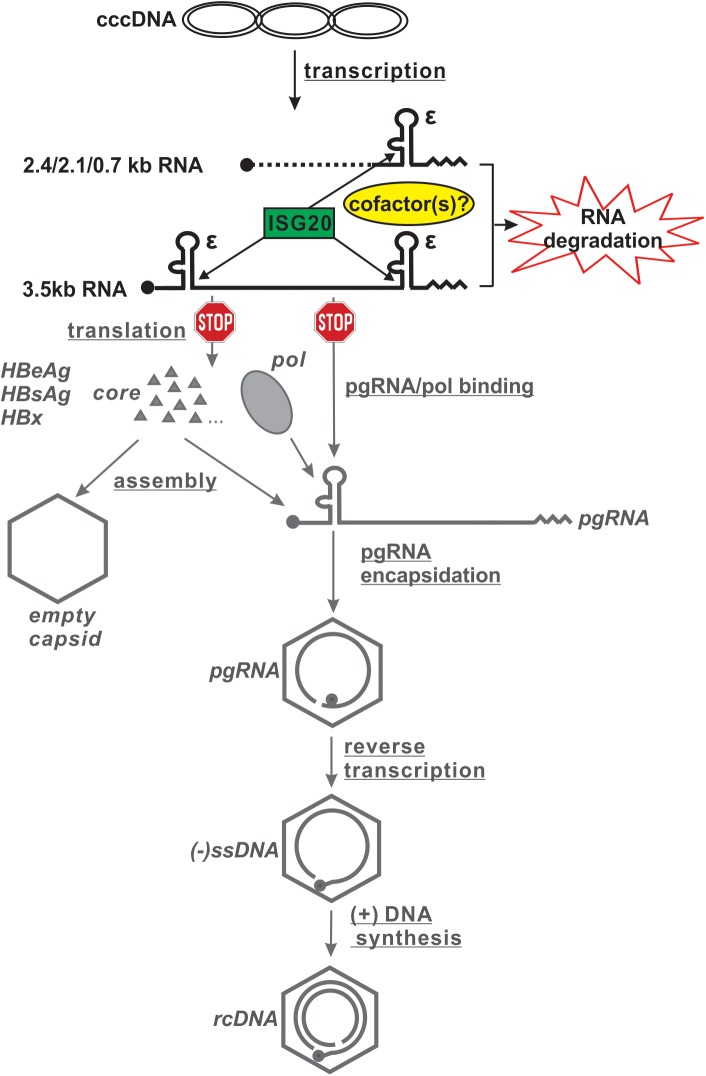
The working model of ISG20-mediated antiviral effect on HBV replication. The major viral intermediates and products at each major HBV replication steps are illustrated. cccDNA (or other HBV transcription template)-derived 3.5kb RNA (including precore mRNA and pgRNA) and other shorter subgenomic RNA species (2.4/2.1kb surface mRNA and 0.7kb X mRNA) are aligned to show the location of ε on different RNA species. The black circle dots indicate the 5’ cap of mRNA, the zigzag lines represent the polyA tails. ISG20 is shown as a rectangle box and its targeting sites on HBV RNA are indicated by arrowheads. As a consequence of ISG20-mediated HBV RNA degradation, the illustrations and labels of viral proteins/antigens, pgRNA encapsidation and DNA replication are shown in gray color schemes. The solid gray triangles indicate capsid proteins, viral polymerase is shown in an oval shape before ε binding and then a gray circle dot in the nucleocapsids after pgRNA encapsidation.

In summary, we report here that ISG20 functions as a 3’-5’ exoribonuclease to degrade HBV RNA, by which serves as an intrinsic host restriction factor for HBV infection. The selectivity of ISG20 for HBV RNA is determined by a unique viral ribonucleotide string that shapes a stem-loop structure (ε) which exists in all HBV RNA species. ISG20 directly binds to the lower stem of ε through the C-terminal Exo III domain, and such protein-RNA interaction, possibly with the assistance of a host cofactor(s), will lead to the major antiviral action of ISG20, which is the enzymatic activity dependent HBV RNA degradation. Subsequently, viral protein translation and DNA replication will be inhibited (Fig 14). In addition, under certain circumstance that the enzymatic activity of ISG20 could be inhibited (i.e. the ISG20^D94G^ mutant), the binding of ISG20 to 5’ ε may remain antiviral by competing the binding of viral polymerase with ε and blocking the subsequent pgRNA encapsidation and reverse transcription.

It is well documented that, during acute HBV infection, the activation of host T-cell responses plays a pivotal role in clearing virus from hepatocytes *via* a noncytolytic mechanism, which is largely attributed to the antiviral and proinflammatory cytokines produced by the infiltrating immune cells, such as IFN-γ and TNF-α [32, 33]. To date, IFN-α is the only available immunotherapy for treatment of chronic hepatitis B [34]. Besides serving as an immunomodulatory agent to regulate the host adaptive immunity against HBV, the intracellular antiviral activity of IFN-α is mediated by an array of ISGs. In search of intrahepatic ISGs associated with immune clearance of HBV, a growing body of evidence implies that ISG20 may be involved in IFN mediated inhibition of HBV, including 1) the upregulation of ISG20 in HBV transgenic mouse hepatocyte cell lines in which the HBV replication was inhibited by IFN [19]; 2) the elevation of ISG20 level in viral clearance phase of an acutely HBV infected Chimpanzee [35]; and 3) the upregulation of ISG20 in chronic hepatitis B patients who responded well to IFN-α therapy [21]. Consistent with the above findings, we found that ISG20 can be highly induced in hepatocytes *in vitro* by all three types of interferons, and it plays a significant role in IFN-α elicited antiviral effects on HBV infection and replication (Figs 1, 4 and 5). Considering that HBV RNA are essential genetic components for viral replication and gene expression in its life cycle, upregulation of ISG20 expression by cytokines to eliminate HBV RNA is a very economical and efficient way in host defense mechanisms. In addition, our study also expands the antiviral spectrum of ISG20 to a DNA virus. Considering that current HBV therapy with nucleoside analogues is ineffective against viral protein expression and cannot efficiently promote HBeAg or HBsAg seroconversion [34], it is thus envisioned that the ISG20-based antiviral therapy could be developed in the future to reduce both viremia and antigenemia in CHB patients.

While its cellular function remains elusive, ISG20 has been previously shown to inhibit a number of RNA viruses, including VSV, influenza viruses, HIV-1, picornaviruses (EMCV, HAV), and flaviviruses (HCV, WNV, and bovine viral diarrhea virus (BVDV)) [16–18, 36, 37]. The previously reported antiviral effects of ISG20 on these RNA viruses are all dependent on its exonuclease activity, though the degradation of viral RNA directly catalyzed by ISG20 has not been experimentally confirmed. However, RNA degradation might not be the only mechanism of ISG20’s antiviral action. For instance, *Zhou et al* found that ISG20 did not promote the degradation of a replication-defective HCV RNA genome in Huh7.5 cells [17]; and *Qu et al* observed that ISG20 inhibits Influenza A Virus replication through interaction with viral nucleoproteins [37]. Furthermore, not all the RNA viruses are susceptible to ISG20, as severe acute respiratory syndrome coronavirus (SARS-CoV) has shown resistance to ISG20 in cell cultures [17], indicating that there is viral specific factor(s) to determine the antiviral specificity of ISG20. Here, we clearly demonstrated that ISG20 is able to promote HBV RNA degradation in cell cultures, and such effect is dependent on the protein’s exonuclease activity (Figs 2–6). During the preparation of this manuscript, Leong *et al* also reported that ISG20 inhibited HBV replication in cell cultures and in hydrodynamic injected mouse liver *via* exoribonuclease-dependent degradation of viral RNA, which is largely consistent with our results, but their study did not touch on the molecular mechanism for the selective targeting of HBV RNA by ISG20 [38]. In our study, we further mapped the sequence element responsible for ISG20-mediated HBV RNA degradation to ε region in HBV RNA genome (Figs 7–9), and confirmed the direct binding of ISG20 to ε in EMSA assay (Fig 10). To the best of our knowledge, this is the first report revealing that the RNA sequence/structure-specific recognition is required for ISG20 to degrade its viral RNA substrate. Such characteristic of ISG20 makes it mechanistically distinct from RNase L, a well-known antiviral ISG that, upon activation, nonspecifically destroys both viral and cellular RNA in cells [39]. In this regard, ISG20 represents a novel class of cellular antiviral ISGs, which is reminiscent of the sequence-specific antiviral RNAi system [40], but ISG20 targets viral RNA directly without the help of small guide RNA.

HBV RNAs are typical mRNAs produced by host RNA polymerase II, all of which possess 5’ cap and a 3' poly (A) tail similar to cellular mRNAs [41]. Despite of the sequence difference with host mRNA, the ε is a unique structure signature of HBV RNA, which serves as pgRNA packaging signal and the priming site for interacting with viral polymerase [42]. It is thus conceivable that ISG20 recognizes ε as a “non-self” target to initiate its antiviral function. In line with this, it has been recently reported that host innate sensor RIG-I binds to ε to trigger IFN-λ production [43]. In addition, we further determined that the bottom 4bp of lower stem of ε is the minimal responsive element of ISG20, as the removal of such sequence from HBV RNA made ISG20 lose both the ε binding activity *in vitro* and HBV RNA reduction effect in cell cultures (Fig 11). These sequence and structure information could serve as a consensus motif to predict ISG20-responsive RNA elements in other viruses, and perhaps host RNAs as well.

Despite acting as a host antiviral factor, the other potential functions of ISG20 in regulating host cellular function remain obscure. Since ISG20 targets viral mRNA for destabilization, it is plausible that they may also alter the stability of certain cellular mRNA to regulate host gene expression. For example, the observed enhancement of HBV S1 promoter activity by ISG20 may be due to the ISG20-mediated upregulation of host transcription factor(s) required for S1 promoter activity or downregulation of negative regulator(s) of S1 promoter (S5 Fig). This notion is also supported by previous observations that ISG20-mediated antiviral effect on HCV was not through inhibition of HCV RNA stability or translation, indicating that ISG20 might degrade the mRNA of a host factor(s) essential for HCV replication [17]. More interestingly, considering that ISG20 favors short RNAs with stem-loop structures for affinity binding, it is also possible that ISG20 potentially targets host microRNA (miRNA) precursors (pri-miRNA and pre-miRNA) [44] and long non-coding RNA (lncRNA) [45], which may contain ε-like stem-loop structures, to regulate miRNA processing and lncRNA stability, respectively. In addition, it is known that IFNs regulate cellular gene expression primarily at the mRNA level [46], so it is possible that ISG20, as a IFN-inducible RNase, may partly mediate the action of IFN through altering the host mRNA stability. We are currently conducting RNA-seq analysis to search for potential host RNA substrates of ISG20.

ISG20 has three predicted exonuclease domains (Exo I-III) with Exo II being validated as the major enzymatic domain [14]. We found that the catalytic site (D94) in Exo II of ISG20 was essential for HBV RNA reduction, but the ISG20^D94G^ mutant retained RNA binding activity and inhibited HBV pgRNA encapsidation through competing the binding of HBV polymerase to ε (Figs 6–8). Although such antiviral effect may be just minor in the context of wildtype ISG20-mediated HBV RNA degradation, this serendipitous finding led us to discover the binding property of ISG20 with HBV RNA (Fig 10). Interestingly, the minimal binding site on ε for ISG20 is located at the bottom 4bp of the stem-loop (Fig 11), which is dispensable for HBV polymerase to bind ε [47], indicating that the inhibition of polymerase binding by ISG20 is due to ISG20/ε interaction-induced steric hindrance and/or conformational shift of ε. In addition, the domain of ISG20 for ε binding was mapped to the Exo III region (Fig 12), suggesting that the substrate interaction and degradation domains of ISG20 are separated. This is consistent with the reported crystal structure analysis of ISG20, which predicted that the amino acid composition of Exo II is unfavorable for RNA binding [30]. However, no classical RNA binding motif was found in the Exo III domain or the entire ISG20 sequence by computational prediction (http://www.rcsb.org/pdb/protein/Q96AZ6). ISG20 has been shown to interact with host U1/2/3 small nucleolar RNAs (snoRNAs) [48], which also have stem-loops, further indicating that ISG20 may possess a novel binding motif for such kind of double stranded RNA secondary structures including HBV ε.

We have previously identified the host zinc-finger antiviral protein (ZAP) as a restriction factor of HBV by promoting HBV RNA degradation [10]. ZAP exerts its antiviral activity against HBV with certain features similar to ISG20, specifically, both ZAP and ISG20 interact with HBV RNA for RNA degradation in a ε-dependent manner. Since ZAP does not possess nuclease activity [49], our initial hypothesis was that ZAP might recruit ISG20 to ε for RNA degradation. However, co-immunoprecipitation assay did not reveal any detectable association between ZAP and ISG20 in the absence or presence of HBV RNA (S10 Fig), indicating that ZAP and ISG20 work independently to target HBV ε for their antiviral functions, and host cells have evolved multiple mechanisms to recognize the “non-self” HBV ε as an antiviral sensing signal.

It is worth to note that ISG20 is a 3’-5’ exoribonuclease and the ε locates near the 3’ end of all HBV RNAs and there is an additional 5’ copy in precore mRNA and pgRNA [14, 50], we therefore speculate that the binding of ISG20 to ε is an initial substrate sensing step, and RNA topology changes and/or ISG20 translocation may be requires for subsequent RNA degradation from 3’ end of HBV RNA. In addition, since ISG20 could single-handedly degrade poly(rA) oligo in the *in vitro* degradation assay (Fig 13B), whether the poly(A)-specific ribonuclease (PARN) is required to remove the poly(A) tail in ISG20-mediated HBV mRNA degradation reaction awaits further investigations. Interestingly, our *in vitro* RNA degradation assay demonstrated that ISG20 efficiently degrades single-stranded RNA but not the ε RNA substrate (Fig 13), indicating that additional host factor(s), likely a RNA helicase, is required to unwind the double-stranded region of ε for ISG20 to catalyze RNA degradation. Therefore, we have embarked on the identification of cofactors in ISG20 degradesome complex by proteomic approach.

Taken together, the phenotypic and mechanistic characterizations of ISG20-mediated antiviral effect on HBV replication presented in current study not only shed light on ISG20 biology and virus-host interaction, but also provide insight into development of ribonuclease-based antiviral therapy for treatment of hepatitis B.

## Materials and methods

### Cell cultures, RNAi, and drugs

Human hepatocyte-derived HepG2 and Huh7 cells were maintained in DMEM/F12 medium (Mediatech) supplemented with 10% fetal bovine serum, 100 U/ml penicillin and 100 μg/ml streptomycin. HepDES19 cells were maintained in the same way as HepG2, but with the addition of 1 μg/ml tetracycline (tet) and 400 μg/ml G418 [24]. To initiate HBV replication in HepDES19 cells, tet was withdrawn from the culture medium and the cells were cultured for the indicated time. Primary human hepatocytes were purchased from Triangle Research Labs and cultured according to supplier’s protocol. HepG2-NTCP12 cells were maintained as previously described [26]. Endogenous ISG20 expression in HepG2-NTCP12 cells was stably knocked down by transduction with ISG20 shRNA lentiviral particles (Santa Cruz Biotechnology) per the manufacturer’s directions. The transduced cells were selected with 3 μg/ml puromycin, the antibiotics-resistant cells were pooled and expanded into cell line, namely HepG2-NTCP12 shISG20. Control known down cell line was generated by transduction with control shRNA lentiviral particles (Santa Cruz Biotechnology) and designated HepG2-NTCP12 shcontrol. Control siRNA and ISG20 siRNA were purchased from Santa Cruz Biotechnology for transient knock down experiments. Recombinant human IFN-α2a, IFN-γ, and IFN-λ1 were purchased from PBL Biomedical Laboratories.

### Plasmids for eukaryotic expression

HBV (genotype D, subtype ayw) replication competent plasmids, pHBV1.3 and pCMVHBV, in which the transcription of viral pgRNA is governed by authentic HBV core promoter and the human cytomegalovirus immediate-early (CMV-IE) promoter, respectively, were described previously [4, 10, 51, 52]. The plasmid pTREHBVDES transcribes pgRNA under the control of tetracycline-inducible CMV promoter, it has been previously used together with plasmid pTet-off (Clontech) to generate the HepDES19 stable cell line [24]. pHBV1.3ΔC is a 1.3mer HBV plasmid with mutation of the start codon (ATG to ATA) of core protein open reading frame (ORF) [53] (provided by Dr. Robert Lanford). pHBV1.3ΔP is a 1.3mer HBV plasmid with start codon mutation (ATG to ACG) of pol ORF to abolish pol expression but without changing the amino acid sequence of the overlapped core protein. Plasmid pCMVHBVΔCΔP contains start codon mutations of core (ATG to CTG) and pol (ATG to ACG) ORF in pCMVHBV backbone to block the expression of core and pol, respectively. pCMVHBV-Y63D bears a Y63D substitution in the reverse transcriptase domain of HBV pol gene to block DNA synthesis but still allows pgRNA encapsidation [54] (Provided by Dr. Jianming Hu). Plasmid pCMV-FLAG-Pol, which expresses HBV Pol with three copies of the FLAG epitope at the N terminus by CMV promoter [55], was provided by Dr. Wang-Shick Ryu and used in cell transfections. Plasmid pcDNA-3FHP, another HBV polymerase expression construct that expresses a 3×FLAG-tagged HBV polymerase (HP) under T7 promoter in pcDNA backbone [56], was provided by Dr. Jianming Hu and used in *in vitro* translation of 3F-HP. Plasmid pLMS expresses 2.4kb and 2.1kb HBV surface mRNA under the control of authentic viral surface promoters (S1 and S2) [57] (provided by Dr. Youhua Xie). Plasmid pMS expresses 2.1kb HBV surface mRNA under the control of a CMV promoter, and pMSΔ4bp was constructed by removing four nucleotides from the bottom right arm of the lower stem of ε sequence in pMS. The pgRNA internal deletion clones (pgID-1 to pgID14) and terminal redundancy (TR) deletion clones (pg-Δ5TR, pg-Δ3TR, pg-Δ5/3TR), and luciferase reporter plasmids (En/Cp-Luc, S1-Luc, S2-Luc, En/Cp-TR-Luc, En/Cp-Luc-TR, and En/Cp-TR-Luc-TR) were constructed previously [10].

Plasmids expressing N-terminal FLAG-tagged wildtype ISG20 (F-ISG20) and ISG20^D94G^ (F-ISG20^D94G^) under the control of CMV promoter were described previously [36]. F-ISG20ΔExoIII was derived from F-ISG20 by deleting the coding sequence for ExoIII domain of ISG20. Plasmid HA-ISG20^D94G^ that expresses HA-tagged ISG20^D94G^ was constructed by Q5 Site-Directed Mutagenesis Kit (New England Biolabs) to replace the FLAG-tag sequence in F- ISG20^D94G^ with HA-tag. Plasmid HA-ZAPS expresses the HA-tagged ZAP-S protein in eukaryotic cells [10].

### Cell transfection

Cells seeded in the collagen coated plate were transfected with indicated plasmid(s) or siRNA by Lipofectamine 2000 (Life Technologies) according to the manufacturer’s directions.

### Reporter assay

HepG2 cells in 96-well-plate were transfected with promoter reporter plasmid plus vectors expressing gene of interest by Lipofectamine 2000. pRL-CMV *Renilla* luciferase reporter plasmid was cotransfected for normalization of transfection efficiency. For each transfection, empty control plasmid was added to ensure that each transfection receives the same amount of total DNA (200 ng/well). Three days after transfection, luciferase activities were measured by the dual luciferase assay kit (Promega) and BioTek Synergy 2 Multi-Mode Reader.

### HBV infection and HBcAg immunofluorescence

HepG2-NTCP12 shcontrol and shISG20 cells were spinoculated with HBV virion particles derived from HepDE19 cells at 100 virus genome equivalent (vge)/cell according to a published protocol [26]. Six days post infection, cells were fixed and subjected to HBcAg immunofluorescence as described previously [26].

### HBV RNA/DNA analyses

Total cellular RNA, Hirt DNA, cytoplasmic encapsidated HBV pgRNA and core DNA were extracted as described previously [10, 24, 51, 58, 59].

For HBV RNA qPCR assay, DNase I-treated total cellular RNA was used to generate cDNA by SuperScript III Reverse Transcriptase (Life Technologies). Real-time PCR was performed with SYBR Green Master (Roche) and the LightCycler 96 System (Roche) for detecting HBV total RNA by using HBV specific primers targeting the 3’ overlapping region of all the HBV RNA species (forward primer: 5’-ACTCTCTCGTCCCCTTCTCC-3’, reverse primer: 5’-GGTCGTTGACATTGCAGAGA-3’). The relative expression levels of HBV RNA were normalized to β-actin from the same samples.

For HBV RNA Northern blot analysis, total cellular RNA or encapsidated pgRNA samples were resolved in 1.5% agarose gel containing 2.2 M formaldehyde and transferred onto Hybond-XL membrane (GE Healthcare). For DNA Southern blot analysis, Hirt DNA or HBV core DNA samples were resolved by electrophoresis into a 1.5% agarose gel and blotted onto Hybond-XL membrane. Membranes were probed with either α-^32^P-UTP (800 Ci/mmol, Perkin Elmer) labeled plus-strand-specific (for Northern blot hybridization) or minus-strand-specific (for Southern blot hybridization) full-length HBV riboprobe and exposed to a phosphorimager screen. Hybridization signals were quantified with QuantityOne software (Bio-Rad).

### HBV capsid gel assay

The cytoplasmic HBV capsid particles were resolved in native agarose gel by electrophoresis and transferred onto nitrocellulose membrane, followed by capsid enzyme immunoassay assay (EIA) using antibodies against core (DAKO) and *in situ* HBV DNA hybridization as described previously [60].

### Western blot assay

Whole cell lysate samples prepared by Laemmli buffer was resolved in a 4%-12% gradient SDS-PAGE and proteins were transferred onto Immobilon PVDF-FL membrane (Millipore). The membranes were blocked with Western Breeze blocking buffer (Life Technologies) and probed with antibodies against ISG20 [16], FLAG-tag (Sigma, clone M2), HA-tag (Covance, clone 16B12), or β-actin (Millipore). Bound antibodies were revealed by IRDye secondary antibodies. The immunoblot signals were visualized and quantified with the Li-COR Odyssey system.

### ELISA

HBeAg and HBsAg in culture fluid were detected by HBeAg ELISA kit (Autobio Diagnostics) and HBsAg ELISA kit (Abazyme) following the manufacturer’s instructions.

### ISG20 and HBV RNA co-immunoprecipitation

HepG2 cells were cotransfected with pCMVHBVΔCΔP and control vector, FLAG-tagged wildtype or D94G mutant ISG20 in 60 mm dishes and maintained for 4 days. The harvested cells were lysed on ice with cell lysis buffer containing 1% NP-40, 10 mM Tris.HCl (pH 7.5), 1 mM EDTA, 50 mM NaCl, 8% sucrose, and 1 U/μl of RNasin Plus RNase Inhibitor (Promega). After centrifugation to remove the cell debris, the clarified cell lysates were incubated with EZview Red Anti-HA or Anti-FLAG Affinity Gel (Sigma-Aldrich) at 4°C for 2 h with gentle rotation. The beads were spun down and resuspended gently with rinse buffer (10 mM Tris.HCl (pH 7.5), 1 mM EDTA, 50 mM NaCl, and 1 U/μl of RNasin Plus RNase Inhibitor) for three times at 4°C. The pelleted beads were subjected to RNA extraction with TRIzol, and protein sample preparation with Laemmli buffer. Immunoprecipitated ISG20 protein and HBV RNA were analyzed by Western blot and Northern blot assays, respectively. In terms of the RNA binding competition assay for ISG20 and HBV Pol, 2 μg of pCMVHBVΔCΔP was cotransfected with either 1 μg of control vector or 1 μg of pCMV-FLAG-Pol and with increased amount of HA-ISG20 D94G as indicated in Fig 8. FLAG or HA immunoprecipitation was performed with the same protocol described above and the associated HBV RNAs were visualized *via* Northern blot using ^32^P radiolabeled HBV RNA specific riboprobe.

### Recombinant His-ISG20 proteins expression and purification

The ORF of wildtype ISG20 and ISG20^D94G^ mutant were PCR amplified from plasmid F-ISG20 and F-ISG20^D94G^, respectively, and cloned into pRSET A prokaryotic expression vector (Thermo Fisher Scientific) under the T7 promoter at BamHI and EcoRI sites to generate plasmid expressing His-tagged ISG20 (His-ISG20) and ISG20^D94G^ (His-ISG20^D94G^). ISG20 deletion mutant His-ISG20ΔExoI, His-ISG20ΔExoII, and His-ISG20ΔExoIII were derived from His-ISG20 by using Q5 Site-Directed Mutagenesis Kit (New England Biolabs) per the manufacture’s protocol. The protein expression vectors were transformed into *Escherichia coli* BL21(DE3) pLysS Competent Cells (Promega) and the cells were propagated with aeration at 37°C in 0.5 L of SOB broth in the presence of 100 μg/ml Ampicillin to an A_600_ of ∼0.6, followed by adding 1 mM isopropyl-1-thio-β-D-galactopyranoside (IPTG) to induce protein expression at 37°C for 3 hours. The induction of the recombinant proteins were detected *via* SDS-PAGE and Coomassie staining as compared to an uninduced control sample.

The aforementioned His-tagged proteins expressed from bacteria were purified using denaturing conditions. Briefly, the bacterial pellet was resuspended in 0.1 M Na-phosphate, 0.1 M Tris-HCl, 6 M Guanidine-HCl, pH 8.0 with fresh 1 mM PMSF and stirred for 2 h at 4°C to lyse the cells and solubilize the proteins under denaturing condition. The cell extract was centrifuged at 12,000×g for 20 min. The supernatant fraction containing soluble protein was incubated in batch with PerfectPro Ni-NTA Agarose (5PRIME) for 1 h at room temperature. The resin was washed once with 0.1 M Na-phosphate, 0.1 M Tris-HCl, 6 M Guanidine-HCl, pH 6.3 and two times with 0.1 M Na-phosphate, 0.1 M Tris-HCl, 6 M Guanidine-HCl, pH 6.3 with additional 20 mM imidazole. The resin was loaded into the supplied purification column, and the protein was eluted with 0.1 M Na-phosphate, 0. 1M Tris-HCl, 6 M Guanidine-HCl, pH 4.6 and 300 mM imidazole with 1 mM PMSF. The eluted protein was dialyzed against three changes of 1×PBS, 300 mM NaCl, 5% Glycerol with freshly added 1 mM DTT and 0.1 mM PMSF. The soluble proteins were concentrated with Pierce concentrators (9KD cut-off, Thermo Scientific). Bio-Rad Bradford protein assay and Coomassie staining were used to measure the concentration and the purity of the proteins.

### Electrophoretic mobility shift assay (EMSA)

The synthetic HBV ε RNA fragments shown in S1 Table were dissolved in 1×TE buffer (DEPC treated) to a concentration of 1 μg/μl and denatured in 80°C water bath for 5 min, followed by slow cooling down to room temperature for RNA annealing and secondary structures formation. HBV ε RNAs were [γ-^32^P] end-labeling by T4 polynucleotide kinase (New England Biolabs) and purified by Quick Spin Sephadex G25 column (Roche).

The indicated amount of ISG20 proteins were incubated with 100 ng ^32^P-radiolabeled HBV RNAs in the presence of 20 mM HEPES, 100 mM KCl, 1 mM DTT, 0.5 mg/ml BSA, 10% Glycerol and 0.05 μg/μl poly[dI-dC] at 30°C for 30 min to form nucleoprotein complexes. 1 μl of monoclonal anti-polyHistidine antibody (Clone H1029, Sigma) was used for supershifting of the His-ISG20/ HBV ε complex. 1 μg, 2 μg or 4 μg of cold unlabeled HBV ε RNAs were used to compete for binding of the ISG20 protein to 100 ng radiolabeled HBV ε. The nucleoprotein complexes were separated by native 5% PAGE at 200 V in a gel buffer containing 50 mM Tris, 45 mM Boric acid, 1% (v/v) Glycerol for 2 h in the cold room. The gel was fixed in 10% acetic acid and 10% methanol, dried, and visualized by autoradiography.

### HBV Pol *in vitro* translation (IVT) and Pol-ε binding

Expression vector pcDNA-3FHP was used to express 3×FLAG tagged HBV Pol (3F-HP) in TnT Coupled Reticulocyte Lysate System (Promega) by following manufacturer’s instructions. The control IVT reaction was done with empty pcDNA3.1 vector (Invitrogen). One microliter of control or 3F-HP IVT sample was mixed with 1 μl of ^32^ P-radiolabeled ε RNA (100 ng/μl) and 18 μl of TMNK RNA binding buffer (20 mM Tris [pH 7.5], 2 mM MgCl2, 15 mM NaCl, 20 mM KCl, and 4 mM DTT) which has been previously used for HBV pol:ε RNA binding reaction [61]. Then 1% Halt protease inhibitor cocktail (ThermoFisher), 4 μl of RNasin RNase inhibitors (Promega), and 6 μg of yeast tRNA were added and the binding reaction was incubated at 30°C for 1 h. The pol:ε binding complex was revealed by EMSA as described above.

### *In vitro* RNA degradation assay

The ribonuclease assay was performed according to previously published reaction conditions for ISG20 [13]. Briefly, the synthetic RNA oligos (S1 Table) were pretreated as described above to allow secondary structure formation before purification and 5’-^32^P labeling. 100 ng of radiolabeled RNA substrates were incubated with RNase A (New England Biolabs) or purified recombinant His-ISG20 in 10 μl nuclease assay buffer (50 mM HEPES-KOH (pH 7.0), 10% glycerol, 50 mM NaCl, 1 mM MnCl_2_, 0.01% Triton X-100, and 1 mM DTT) at 37°C for 15 min. The reactions were stopped with 10 μl of an 80% Formamide dye solution, and the mixtures were heated at 80°C for 3 min and then fractionated in Novex 10% TBE-Urea denaturing polyacrylamide gel (ThermoFisher), and the gel was dried and subjected to autoradiography.

## Supporting information

S1 Fig Ectopic expression of ISG20 reduced HBV RNA in a dose dependent manner. HepG2 cells in 12-well-plate were cotransfected with 1.2 μg of pHBV1.3 and variable amount of plasmid F-ISG20 (as indicated), control vector was supplied to normalize the total plasmid DNA in each transfection to 1.6 μg. Cells were harvest at day 5 posttransfection and the levels of viral RNA were determined by Northern blot hybridization (top panel). Ribosomal RNAs (28S and 18S) are presented as loading controls. The relative pgRNA level in each sample is expressed as the percentage of RNA of the cells received no F-ISG20 (lane 1). ISG20 expression was confirmed by Western blot using monoclonal antibody against FLAG tag. β-actin expression was presented as protein loading control (bottom panel).
(TIF)

S2 Fig ISG20 overexpression is nontoxic to the cells. HepG2 cells in 96-well-plate were transfected with control empty vector, or pHBV1.3, or pHBV1.3 plus F-ISG20. 5 days later, cell viability was measured by CytoTox-ONE Homogeneous Membrane Integrity Assay, and the relative cell viability values were plotted as percentage of the value from control samples (mean±SD, n = 5).
(TIF)

S3 Fig Antiviral effects of ISG20 on HBV surface mRNA and antigen production. (A) ISG20 overexpression reduces the levels of HBV surface mRNA. HepG2 cells in 12-well-plate were cotransfected with 0.8 μg of pLMS and 0.8 μg of control vector or plasmid F-ISG20. Four days later, HBV surface mRNA (2.4 kb and 2.1 kb in length) were detected by Northern blot hybridization. Results from duplicate experiments are presented. (B) ISG20 overexpression reduces the levels of viral antigens. HepG2 cells in 12-well-plate were cotransfected with 0.8 μg of pHBV1.3 and 0.8 μg of control vector or plasmid F-ISG20. Four days later, the levels of HBeAg and HBsAg in culture supernatant were measured by ELISA. The relative level of HBeAg and HBsAg signals in each sample was plotted as the percentage of the signals from the control samples (mean ± SD, n = 4).
(TIF)

S4 Fig ISG20 does not alter the levels of HBV RNA transcription template. (A) ISG20 overexpression does not reduce the level of transfected HBV plasmid DNA. HepG2 cells in 12-well-plate were cotransfected with 0.8 μg of plasmid pHBV1.3 and 0.8 μg of control vector or plasmid F-ISG20. The cells were harvested at day 5 post transfection, total Hirt DNA was treated by Dpn I and subjected to HBV DNA Southern blot (top panel). During cytoplasmic HBV DNA extraction, DNase I digestion of input HBV plasmid DNA in cell lysates was omitted, and the recovered cytoplasmic DNA samples were treated with Dpn I to digest the bacteria-derived plasmid DNA with Dam methylation, but not the viral core DNA synthesized in eukaryotic cells. The Dpn I-restricted pHBV1.3 DNA fragments migrated at the bottom of the gel were revealed together with HBV core DNA by Southern blot using HBV probe (middle panel). Expression of ISG20 was detected by Western blot with antibodies against FLAG-tag. β-actin served as loading control. (B) Expression of ISG20 reduces HBV RNA in HBV stable cell line. Tetracycline inducible (tet-off) HBV stable cell line HepDES19 cells, which transcribes HBV RNA from the integrated HBV genome, were transfected with control vector or plasmid F-ISG20 in tet-free medium. Four days later, HBV RNA and ISG20 expression were analyzed by Northern and Western blot, respectively.
(TIF)

S5 Fig ISG20 overexpression does not inhibit HBV promoter activity. HepG2 cells in 96-well-plate were co-transfected with reporter plasmid expressing luciferase under the control of HBV core promoter (EnII/Cp), or preS1 promoter (S1), or preS2/S promoter (S2), or CMV-IE promoter, and control vector or plasmid F-ISG20. Cells were lysed two days posttransfection and the relative luciferase activities was plotted as percentage of the luciferase activity from each corresponding control samples (mean±SD, n = 3).
(TIF)

S6 Fig ISG20^D94G^ inhibits pgRNA encapsidation of a replication-defective HBV with polymerase Y63D mutation. Plasmid pCMVHBV-Y63D encodes a replication defective HBV genome due to the mutation of priming site (Y63D) in viral polymerase TP domain, which, upon transfection, arrests HBV replication at pgRNA encapsidation step without subsequent reverse transcription. This plasmid was cotransfected into HepG2 cells with empty vector, or F-ISG20, or F-ISG20^D94G^. 4 days later, viral total RNA, cytoplasmic capsid, encapsidated pgRNA (capsid RNA), capsid DNA, and ISG20 expression were analyzed.
(TIF)

S7 Fig ISG20-mediated HBV RNA degradation does not rely on viral core or pol protein. The core-minus plasmid (pHBV1.3ΔC) or Pol-minus plasmid (pHBV1.3ΔP) was cotransfected into HepG2 cells with either control empty vector or plasmid F-ISG20. 4 days later, HBV total RNA was analyzed by Northern blot.
(TIF)

S8 Fig The ISG20 responsive elements are not within the HBV pgRNA sequence between two TRs. (A) Schematic illustrations of HBV clones that express pgRNA with internal sequence deletions. The deleted regions of the internal deletion clones (pg-ID1 to pg-ID14) are between the indicated 5’ nucleotide positions and a fixed 3’ position at the second Rsr II restriction site (nt1574). (B) Sensitivity of HBV pgRNA with internal sequence deletions to ISG20-mediated RNA reduction. Plasmid pHBV1.3 and the internal deletion clones were transfected into HepG2 cells individually with control plasmid or F-ISG20. Four days later, viral RNA was analyzed by Northern blot.
(TIF)

S9 Fig EMSA of HBV Pol:ε binding. (A) 3×FLAG-tagged HBV pol (3F-HP) was expressed in rabbit reticulocyte lysate (RRL) through *in vitro* translation (IVT). The recombinant Pol protein expression was confirmed by Western blot using FLAG antibody. (B) Control IVT sample or 3F-HP IVT sample was incubated with 100 ng of 5’-radiolabeled ε RNA in TMNK binding buffer, and the Pol-ε RNP complex was detected by EMSA.
(TIF)

S10 Fig ISG20 does not associate with ZAP in cell cultures. HepG2 cells were mock transfected (lane 1), or transfected with HA-ZAPS alone (lane 2), or cotransfected with F-ISG20 and HA-ZAPS (lane 3), or cotransfected with F-ISG20, HA-ZAPS, and pHBV1.3 (lane 4). Five days later, the expression of F-ISG20 and HA-ZAPS was detected by Western blot using antibodies against FLAG-tag and HA-tag, respectively, and β-actin served as loading control (top panels). Then cell lysates were subjected to FLAG or HA immunoprecipitation (IP) and the immunoprecipitated samples were subjected to Western blot (WB) assay with indicated antibodies (bottom panels).
(TIF)

S1 Table Sequence of synthetic RNA oligos. (PDF)
