# Supplementary figures and images for: Interferon-inducible ribonuclease ISG20 inhibits hepatitis B virus replication through directly binding to the epsilon stem-loop structure of viral RNA
Source: PLoS Pathog. 2017 Apr 11;13(4):e1006296. doi: 10.1371/journal.ppat.1006296 (PMC5388505; doi:10.1371/journal.ppat.1006296)

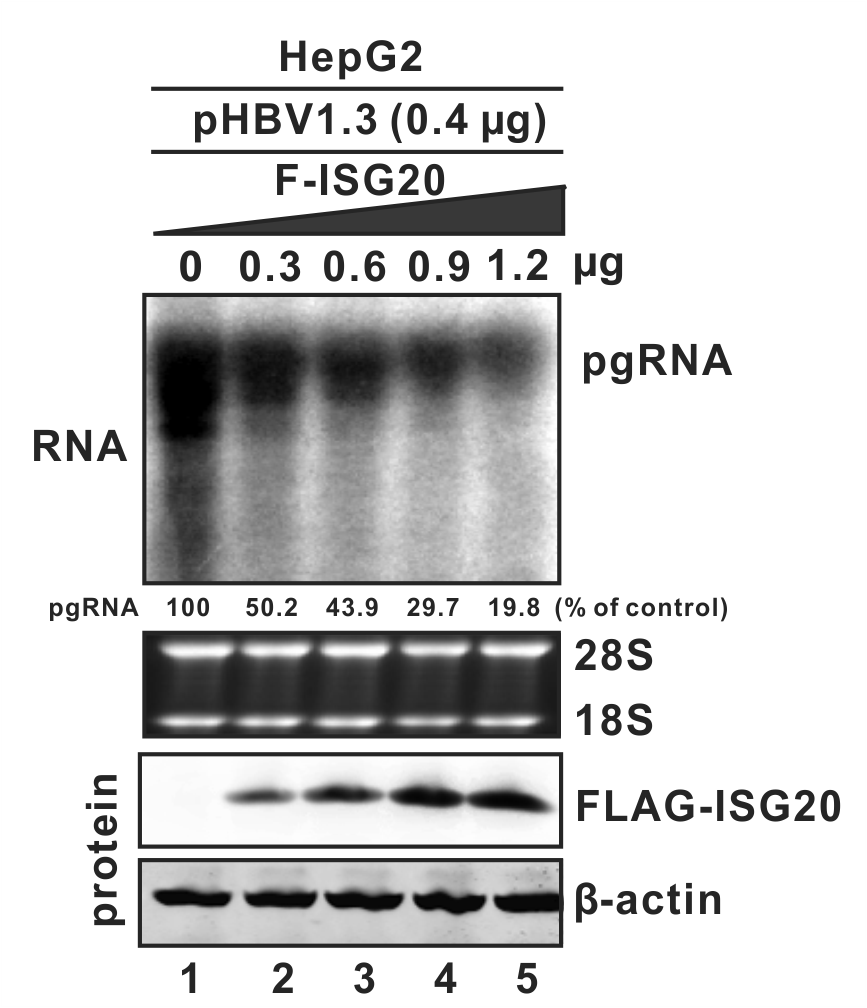

Supplement: S1 Fig — HepG2 cells in 12-well-plate were cotransfected with 1.2 μg of pHBV1.3 and variable amount of plasmid F-ISG20 (as indicated), control vector was supplied to normalize the total plasmid DNA in each transfection to 1.6 μg. Cells were harvest at day 5 posttransfection and the levels of viral RNA were determined by Northern blot hybridization (top panel). Ribosomal RNAs (28S and 18S) are presented as loading controls. The relative pgRNA level in each sample is expressed as the percentage of RNA of the cells received no F-ISG20 (lane 1). ISG20 expression was confirmed by Western blot using monoclonal antibody against FLAG tag. β-actin expression was presented as protein loading control (bottom panel). (TIF) [file ppat.1006296.s001.tif]

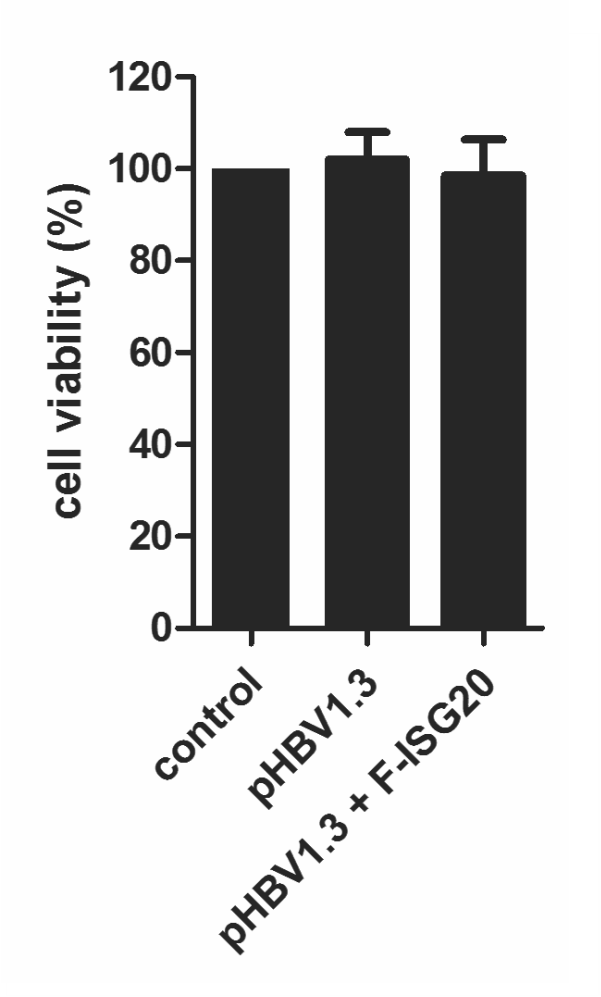

Supplement: S2 Fig — HepG2 cells in 96-well-plate were transfected with control empty vector, or pHBV1.3, or pHBV1.3 plus F-ISG20. 5 days later, cell viability was measured by CytoTox-ONE Homogeneous Membrane Integrity Assay, and the relative cell viability values were plotted as percentage of the value from control samples (mean±SD, n = 5). (TIF) [file ppat.1006296.s002.tif]

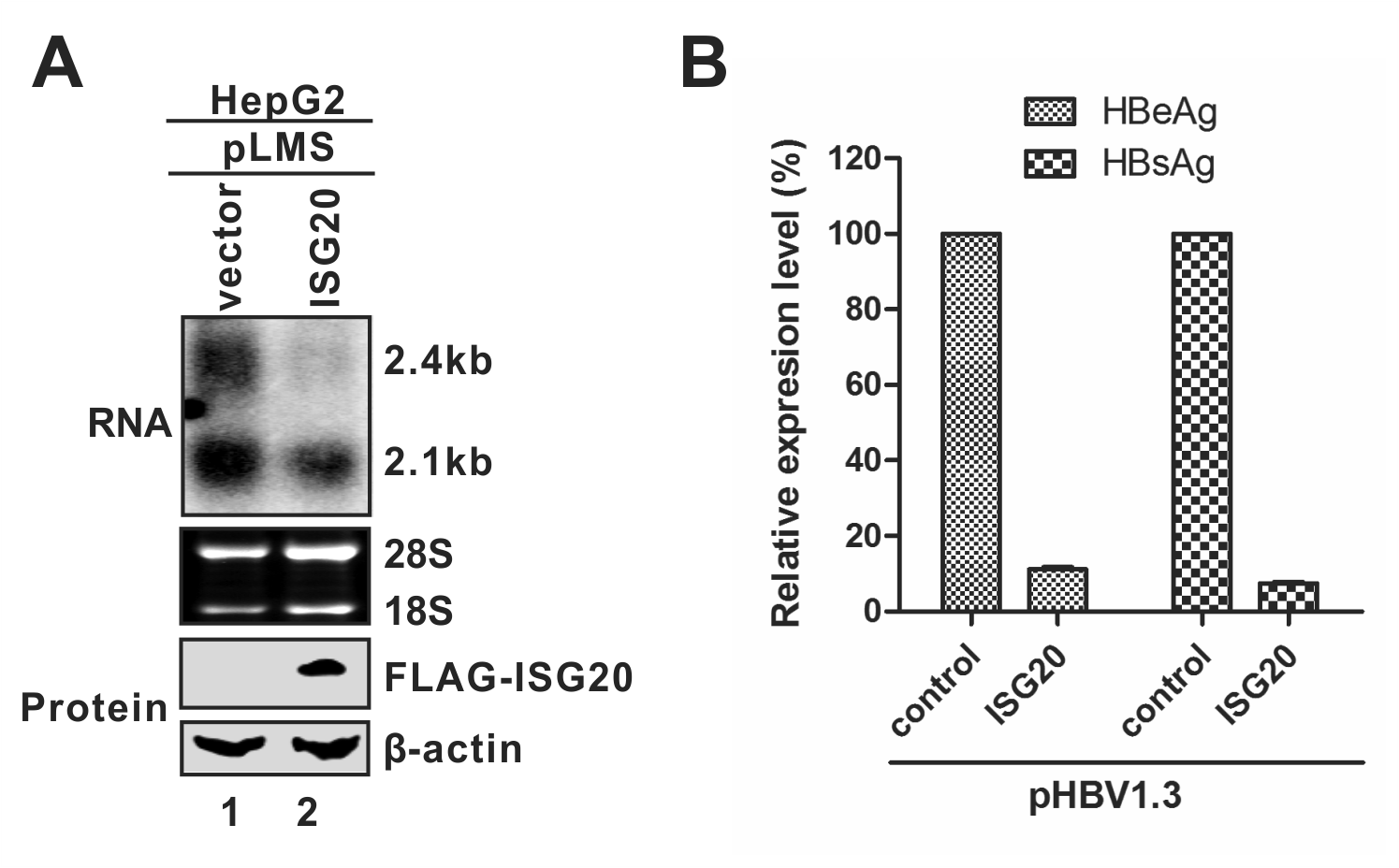

Supplement: S3 Fig — (A) ISG20 overexpression reduces the levels of HBV surface mRNA. HepG2 cells in 12-well-plate were cotransfected with 0.8 μg of pLMS and 0.8 μg of control vector or plasmid F-ISG20. Four days later, HBV surface mRNA (2.4 kb and 2.1 kb in length) were detected by Northern blot hybridization. Results from duplicate experiments are presented. (B) ISG20 overexpression reduces the levels of viral antigens. HepG2 cells in 12-well-plate were cotransfected with 0.8 μg of pHBV1.3 and 0.8 μg of control vector or plasmid F-ISG20. Four days later, the levels of HBeAg and HBsAg in culture supernatant were measured by ELISA. The relative level of HBeAg and HBsAg signals in each sample was plotted as the percentage of the signals from the control samples (mean ± SD, n = 4). (TIF) [file ppat.1006296.s003.tif]

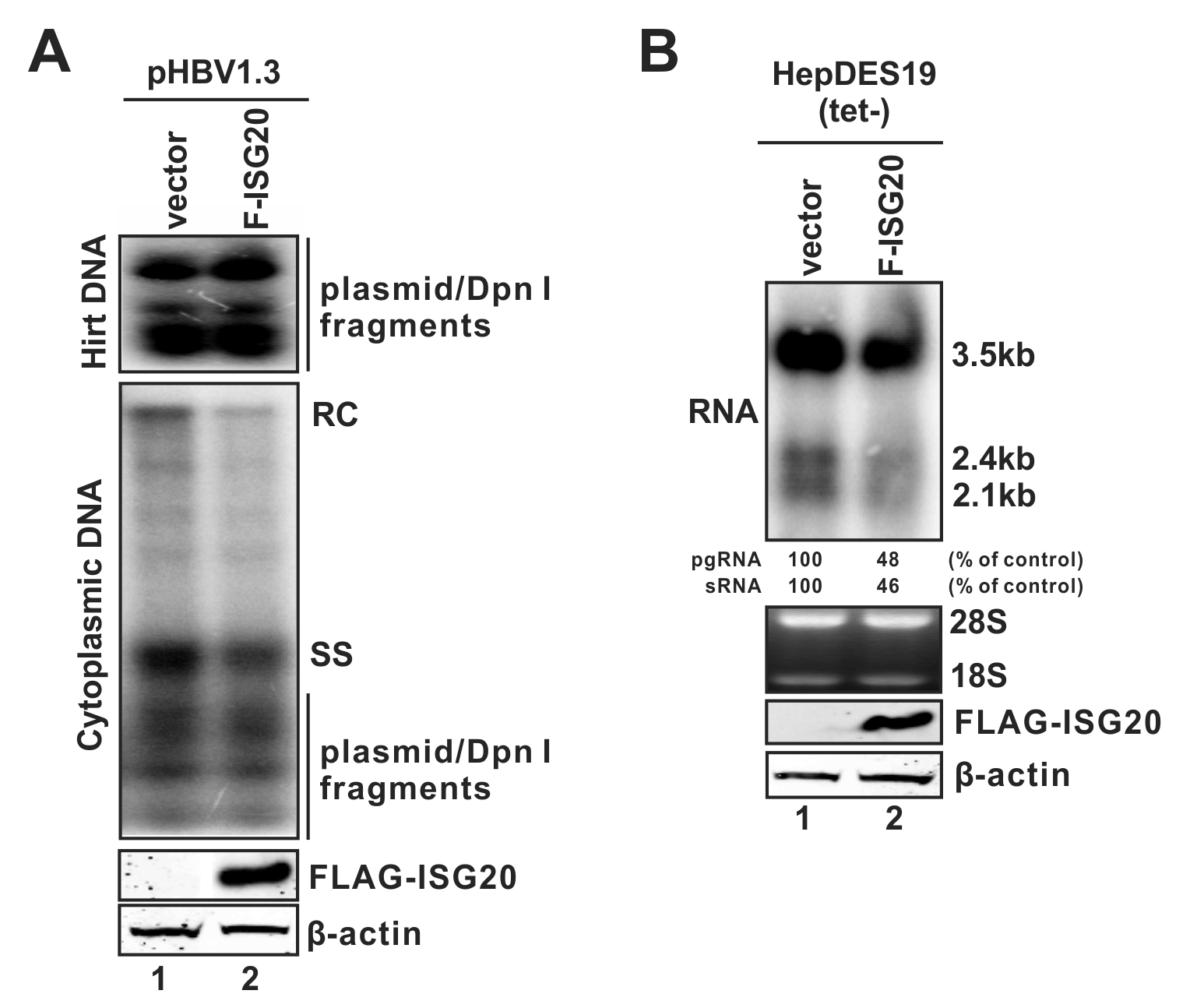

Supplement: S4 Fig — (A) ISG20 overexpression does not reduce the level of transfected HBV plasmid DNA. HepG2 cells in 12-well-plate were cotransfected with 0.8 μg of plasmid pHBV1.3 and 0.8 μg of control vector or plasmid F-ISG20. The cells were harvested at day 5 post transfection, total Hirt DNA was treated by Dpn I and subjected to HBV DNA Southern blot (top panel). During cytoplasmic HBV DNA extraction, DNase I digestion of input HBV plasmid DNA in cell lysates was omitted, and the recovered cytoplasmic DNA samples were treated with Dpn I to digest the bacteria-derived plasmid DNA with Dam methylation, but not the viral core DNA synthesized in eukaryotic cells. The Dpn I-restricted pHBV1.3 DNA fragments migrated at the bottom of the gel were revealed together with HBV core DNA by Southern blot using HBV probe (middle panel). Expression of ISG20 was detected by Western blot with antibodies against FLAG-tag. β-actin served as loading control. (B) Expression of ISG20 reduces HBV RNA in HBV stable cell line. Tetracycline inducible (tet-off) HBV stable cell line HepDES19 cells, which transcribes HBV RNA from the integrated HBV genome, were transfected with control vector or plasmid F-ISG20 in tet-free medium. Four days later, HBV RNA and ISG20 expression were analyzed by Northern and Western blot, respectively. (TIF) [file ppat.1006296.s004.tif]

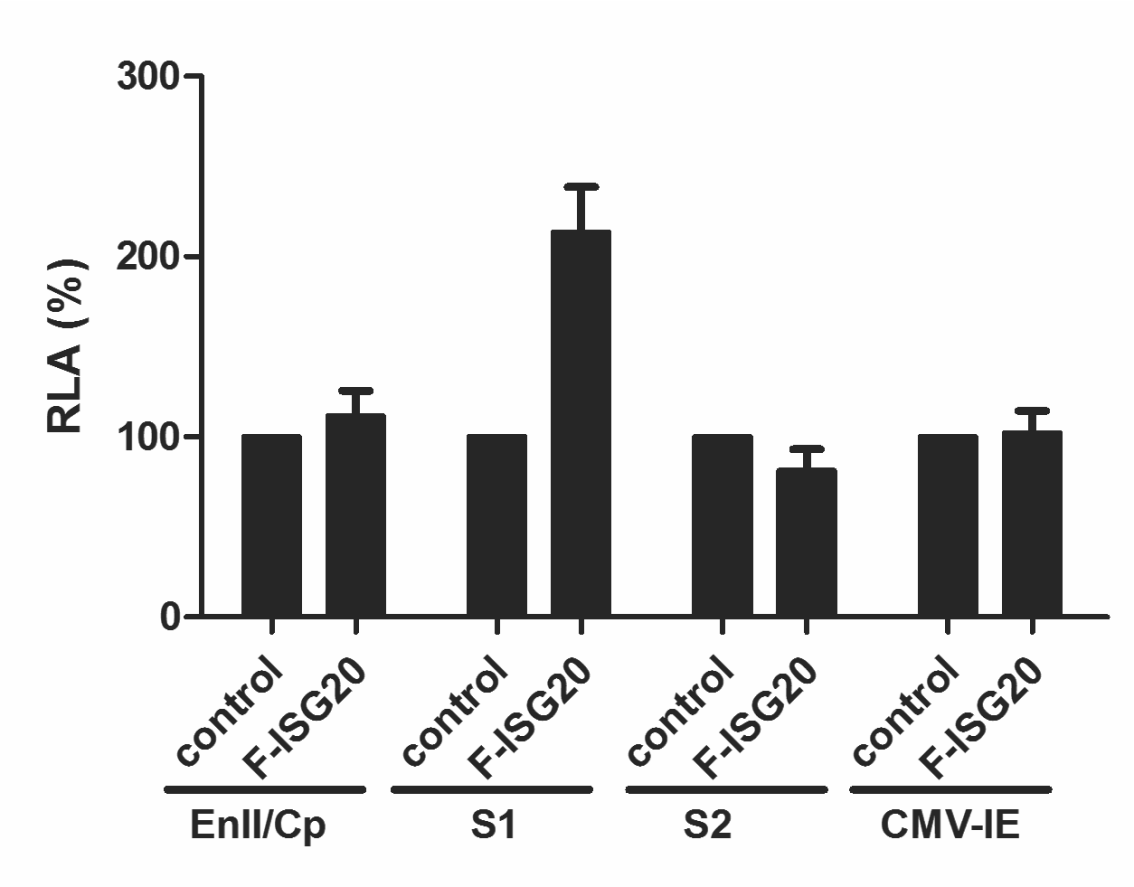

Supplement: S5 Fig — HepG2 cells in 96-well-plate were co-transfected with reporter plasmid expressing luciferase under the control of HBV core promoter (EnII/Cp), or preS1 promoter (S1), or preS2/S promoter (S2), or CMV-IE promoter, and control vector or plasmid F-ISG20. Cells were lysed two days posttransfection and the relative luciferase activities was plotted as percentage of the luciferase activity from each corresponding control samples (mean±SD, n = 3). (TIF) [file ppat.1006296.s005.tif]

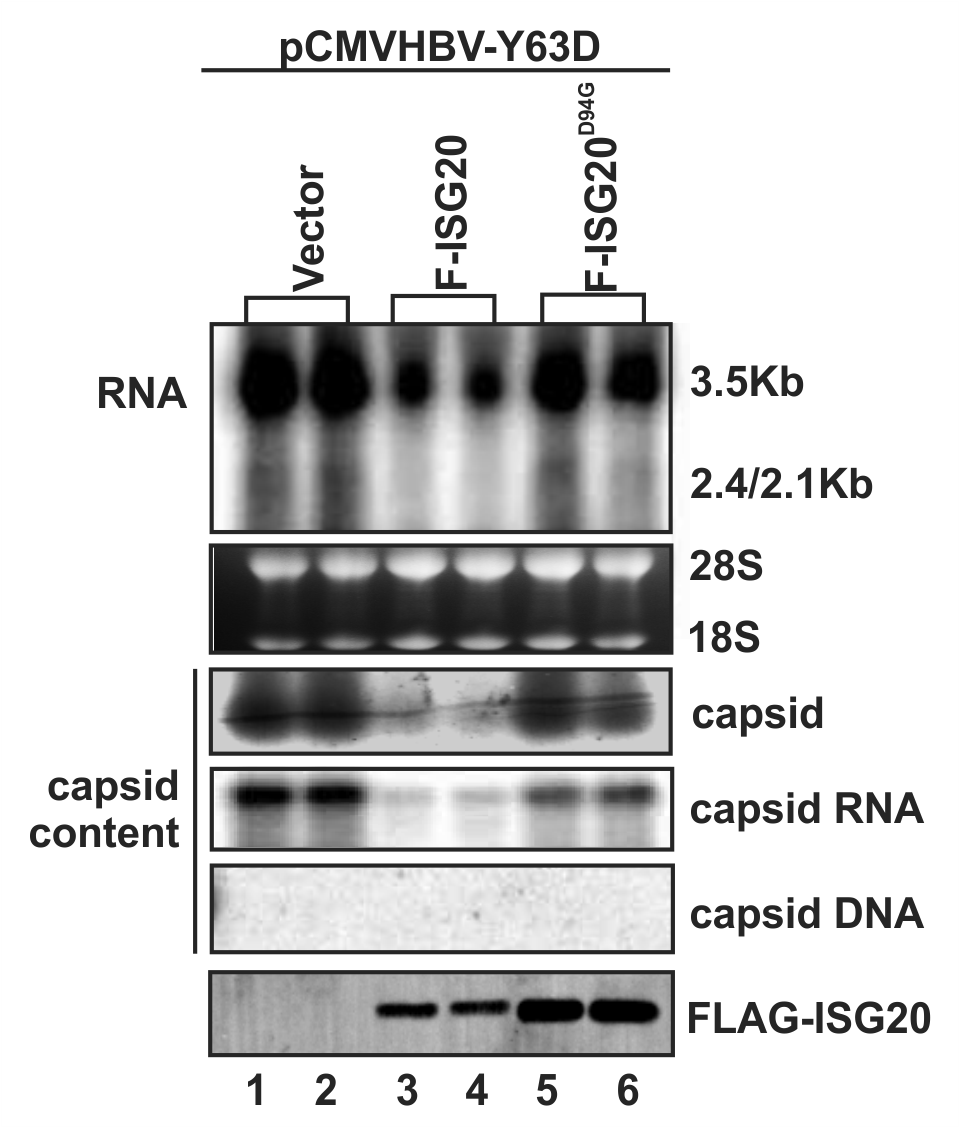

Supplement: S6 Fig — Plasmid pCMVHBV-Y63D encodes a replication defective HBV genome due to the mutation of priming site (Y63D) in viral polymerase TP domain, which, upon transfection, arrests HBV replication at pgRNA encapsidation step without subsequent reverse transcription. This plasmid was cotransfected into HepG2 cells with empty vector, or F-ISG20, or F-ISG20D94G. 4 days later, viral total RNA, cytoplasmic capsid, encapsidated pgRNA (capsid RNA), capsid DNA, and ISG20 expression were analyzed. (TIF) [file ppat.1006296.s006.tif]

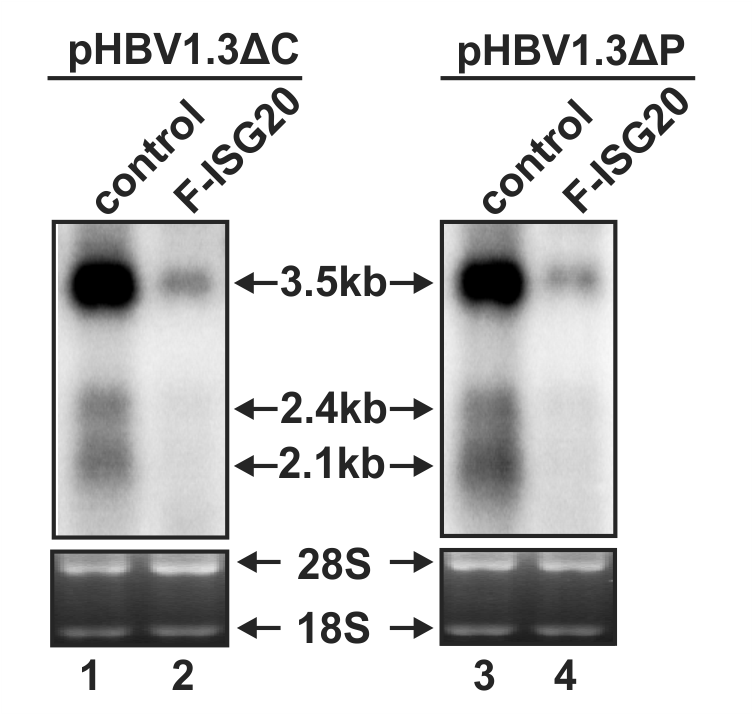

Supplement: S7 Fig — The core-minus plasmid (pHBV1.3ΔC) or Pol-minus plasmid (pHBV1.3ΔP) was cotransfected into HepG2 cells with either control empty vector or plasmid F-ISG20. 4 days later, HBV total RNA was analyzed by Northern blot. (TIF) [file ppat.1006296.s007.tif]

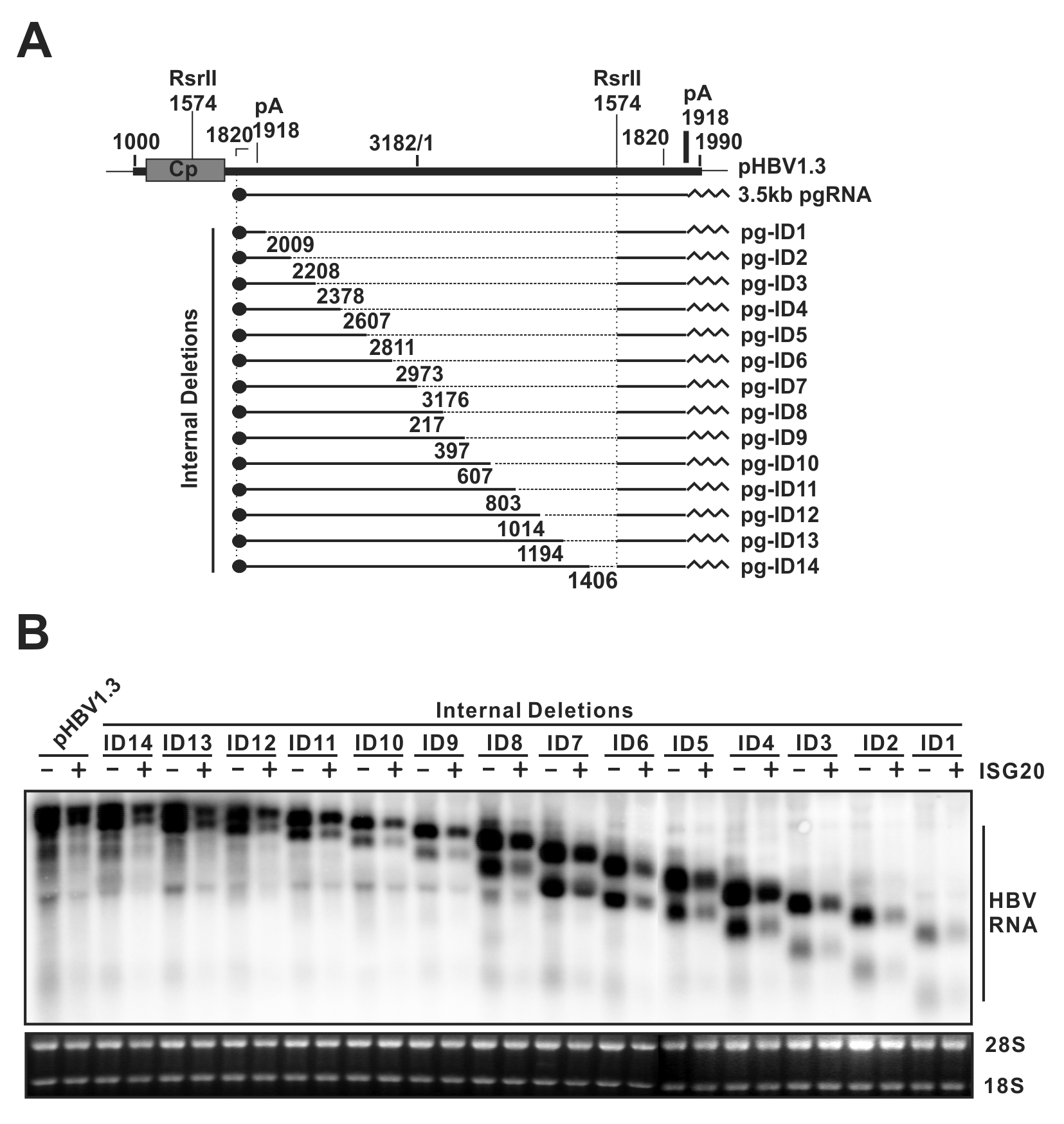

Supplement: S8 Fig — (A) Schematic illustrations of HBV clones that express pgRNA with internal sequence deletions. The deleted regions of the internal deletion clones (pg-ID1 to pg-ID14) are between the indicated 5’ nucleotide positions and a fixed 3’ position at the second Rsr II restriction site (nt1574). (B) Sensitivity of HBV pgRNA with internal sequence deletions to ISG20-mediated RNA reduction. Plasmid pHBV1.3 and the internal deletion clones were transfected into HepG2 cells individually with control plasmid or F-ISG20. Four days later, viral RNA was analyzed by Northern blot. (TIF) [file ppat.1006296.s008.tif]

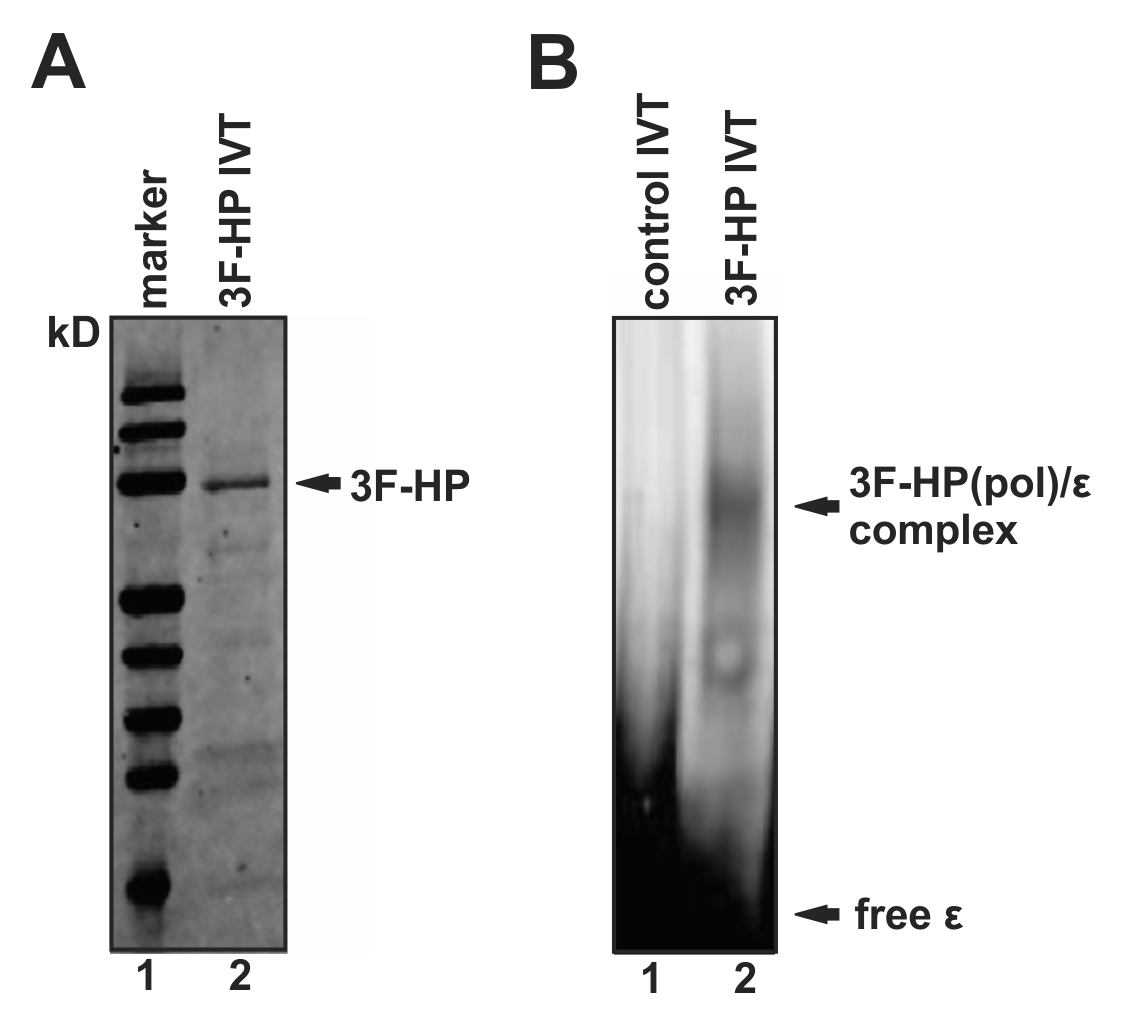

Supplement: S9 Fig — (A) 3×FLAG-tagged HBV pol (3F-HP) was expressed in rabbit reticulocyte lysate (RRL) through in vitro translation (IVT). The recombinant Pol protein expression was confirmed by Western blot using FLAG antibody. (B) Control IVT sample or 3F-HP IVT sample was incubated with 100 ng of 5’-radiolabeled ε RNA in TMNK binding buffer, and the Pol-ε RNP complex was detected by EMSA. (TIF) [file ppat.1006296.s009.tif]

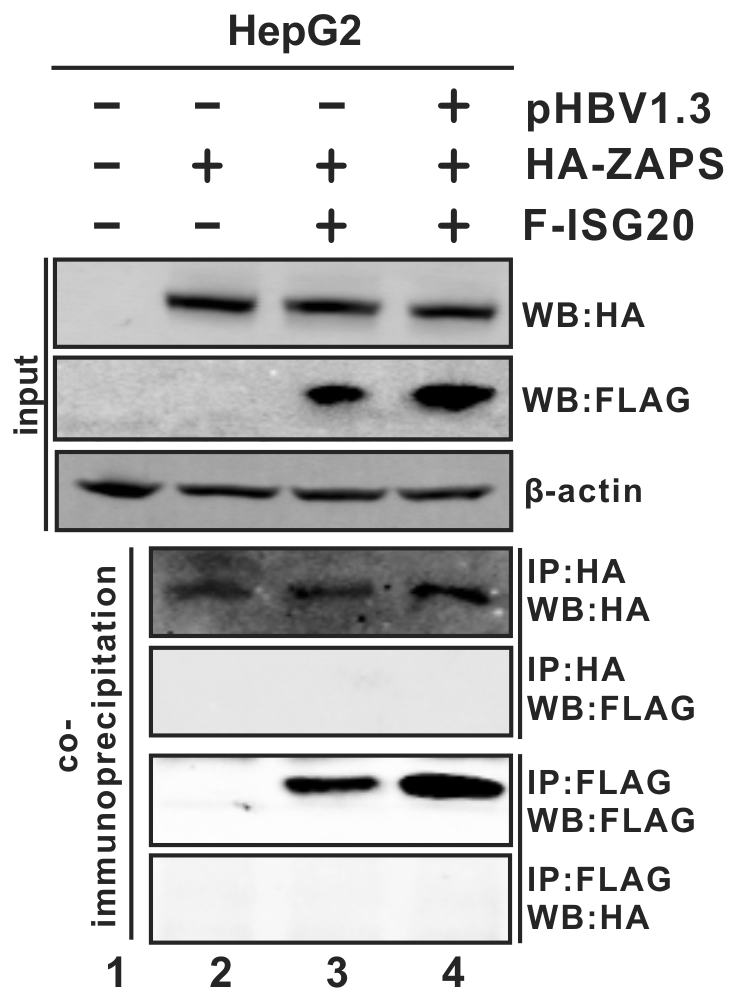

Supplement: S10 Fig — HepG2 cells were mock transfected (lane 1), or transfected with HA-ZAPS alone (lane 2), or cotransfected with F-ISG20 and HA-ZAPS (lane 3), or cotransfected with F-ISG20, HA-ZAPS, and pHBV1.3 (lane 4). Five days later, the expression of F-ISG20 and HA-ZAPS was detected by Western blot using antibodies against FLAG-tag and HA-tag, respectively, and β-actin served as loading control (top panels). Then cell lysates were subjected to FLAG or HA immunoprecipitation (IP) and the immunoprecipitated samples were subjected to Western blot (WB) assay with indicated antibodies (bottom panels). (TIF) [file ppat.1006296.s010.tif]
