# Supplementary material for: Interferon-inducible ribonuclease ISG20 inhibits hepatitis B virus replication through directly binding to the epsilon stem-loop structure of viral RNA
Source: PLoS Pathog. 2017 Apr 11;13(4):e1006296. doi: 10.1371/journal.ppat.1006296 (PMC5388505; doi:10.1371/journal.ppat.1006296)
Supplement: S1 Table — (PDF) [file ppat.1006296.s011.pdf]

Supplement Table 1. Sequence of synthetic RNA oligos.

Name	Sequence (5'→3' orientation)
Full-length ε (FL)	CUUGUUCAUGUCCUACUGUUCAAGCCUCCAAGCUGUGCCUUGGGUGGCUUUGG GGCAUGGACAUC
Upper stem + loop (US+L)	UCAAGCCUCCAAGCUGUGCCUUGGGUGGCUUUG
Lower stem + bulge (LS+B)	CUUGUUCAUGUCCUACUGUUCUGGGGCAUGGACAUC
Lower stem + bulge mutant (LS+Bm)	CUUGUUCAUGUCCUACUAAAAUGGGGCAUGGACAUC
Lower stem Δ4bp + bulge (LSΔ4+B)	CAUGUCCUACUGUUCUGGGGCAUG
Left arm	UGUUCAUGUCCUACUGUUCAAGCCUCCAAG
30-mer poly(rA)	AAAAAAAAAAAAAAAAAAAAAAAAAAAAAA
